# Small molecule inhibitors and CRISPR/Cas9 mutagenesis demonstrate that SMYD2 and SMYD3 activity are dispensable for autonomous cancer cell proliferation

**DOI:** 10.1371/journal.pone.0197372

**Published:** 2018-06-01

**Authors:** Michael J. Thomenius, Jennifer Totman, Darren Harvey, Lorna H. Mitchell, Thomas V. Riera, Kat Cosmopoulos, Alexandra R. Grassian, Christine Klaus, Megan Foley, Elizabeth A. Admirand, Haris Jahic, Christina Majer, Tim Wigle, Suzanne L. Jacques, Jodi Gureasko, Dorothy Brach, Trupti Lingaraj, Kip West, Sherri Smith, Nathalie Rioux, Nigel J. Waters, Cuyue Tang, Alejandra Raimondi, Michael Munchhof, James E. Mills, Scott Ribich, Margaret Porter Scott, Kevin W. Kuntz, William P. Janzen, Mikel Moyer, Jesse J. Smith, Richard Chesworth, Robert A. Copeland, P. Ann Boriack-Sjodin

**Affiliations:** Epizyme, Inc., Cambridge, Massachusetts, United States of America; Cornell University, UNITED STATES

## Abstract

A key challenge in the development of precision medicine is defining the phenotypic consequences of pharmacological modulation of specific target macromolecules. To address this issue, a variety of genetic, molecular and chemical tools can be used. All of these approaches can produce misleading results if the specificity of the tools is not well understood and the proper controls are not performed. In this paper we illustrate these general themes by providing detailed studies of small molecule inhibitors of the enzymatic activity of two members of the SMYD branch of the protein lysine methyltransferases, SMYD2 and SMYD3. We show that tool compounds as well as CRISPR/Cas9 fail to reproduce many of the cell proliferation findings associated with SMYD2 and SMYD3 inhibition previously obtained with RNAi based approaches and with early stage chemical probes.

## Introduction

The human protein methyltransferases (PMTs) constitute a large class of enzymes that play important roles in the post-translational modification of, among other proteins, the histone components of chromatin. By site-specific modification of histone lysine or arginine amino acid side chains, these enzymes effect chromatin structural changes that in turn control programs of gene transcription. The PMTs have garnered significant interest because a number of these enzymes are dysregulated in human diseases, including many oncology indications. Small molecule inhibitors for a number of these enzymes have been reported over the past decade and inhibitors against three PMT targets (DOT1L, EZH2 and PRMT5) have transitioned to clinical trials as therapeutic agents against different human cancers. Within the protein lysine methyltransferase (PKMT) family of enzymes, one branch contains five enzymes that share two highly conserved, functional domains: the catalytic SET domain and the MYND domain; these five enzymes are known as SMYD1, SMYD2, SMYD3, SMYD4 and SMYD5 [1, 2].

Among the SMYD enzymes, SMYD2 and SMYD3 have been implicated as targets for a variety of cancer indications. SMYD2 is overexpressed in tumor types including esophageal squamous carcinoma, bladder and gastric cancers and pediatric acute lymphoblastic leukemia [3–6]. Consistent with its role in tumorigenesis, knockdown of SMYD2 in esophageal, bladder and gastric cancer models is reported to attenuate proliferation in a variety of tissue culture cells [4, 6, 7]. Moreover, mouse models of KRAS-driven pancreatic cancer were shown to be partially dependent on SMYD2 and indicate that genotoxic agents are more effective in the absence of SMYD2 activity. The authors suggest that this effect is due to SMYD2’s regulation of the stress kinase, MAPKAPK3 [8]. In addition, mouse AML models also showed SMYD2 to be a Myc target and were required for MLL-AF9 induced leukemogenesis [9].

SMYD2 inhibitors with varying chemical structures have recently been described [10–13]. Some of the reported compounds were shown to inhibit intracellular methylation of known SMYD2 substrates. Nevertheless, the phenotypic consequences of this inhibition (i.e. cell proliferation) are not yet fully understood. The reported SMYD2 inhibitor LLY-507 has been shown to inhibit esophageal, breast and liver cancer cell line proliferation. However, additional reports (http://www.chemicalprobes.org) indicate that this compound is a potent inhibitor (<1 μM) of several enzymes which could complicate interpretation of the cell proliferation data. In addition, Eggert *et al* [10] reported an equally potent SMYD2 inhibitor, BAY-598, with vastly different phenotypic effects than previously seen for published SMYD2 inhibitors. BAY-598 inhibits SMYD2 with an IC_50_ of 27 nM, inhibits intracellular substrate methylation with an IC_50_ of 58 nM, but has little impact on cell proliferation or induction of apoptosis in cell lines found to be sensitive to LLY-507 [10, 11]. This discrepancy highlights a need for additional, high-quality *in vitro* and *in vivo* probes of SMYD2 with good physicochemical properties and representing chemical structures distinct from those previously described, as well as robust genetic testing of the proliferation effect due to SMYD2 loss.

SMYD3 has also been implicated in a number of human cancers. This enzyme is highly expressed in breast, liver, and colorectal- cancers [14, 15]. Knockdown of SMYD3 by RNAi has been reported to result in decreased cell proliferation in hepatocellular carcinoma, breast, cervical and esophageal cell lines and also in oncogenic KRAS -driven pancreatic cancer and lung adenocarcinoma cell lines [14–26]. In addition, over-expression of SMYD3 in NIH-3T3 cells has been shown to induce a transformed phenotype with enhanced growth rates [15, 19]. More importantly, *in vivo* studies of SMYD3 knockout mice suggest that the enzyme is involved in KRAS driven lung and pancreatic cancer development and in the early stages of liver and colon carcinogenesis [21, 27]. SMYD3 has also been shown to regulate MAPK pathways by methylating MAP3K2 [21]. As a result of many of these studies, several groups have generated small molecule inhibitors of SMYD3 with varying effects on cellular proliferation [28–30].

In the current study we exemplify pharmacophore series for SMYD2 and SMYD3 that show low nanomolar potency against their targets and significant selectivity over all other enzymes tested with favorable physicochemical properties and pharmacological tractability. These inhibitors permeate cancer cells, resulting in intracellular inhibition of the relevant methyl marks for each enzyme. Nevertheless, and in contrast to many of the earlier studies summarized above, these SMYD2 and SMYD3 inhibitors show no impact on the cell proliferation of more than 240 cancer cell lines regardless of genetic or histological background. Similarly, knockout of these genes with CRISPR/Cas9 across 313 cell lines shows no proliferative effects. Based on these findings, we conclude that despite previous observations using RNAi based techniques and early stage chemical probes, SMYD2 and SMYD3 are not required for autonomous proliferation of cancer cells.

## Materials and methods

### Compound synthesis and ADME methods

See Supporting Information S1 File.

### Protein purification

The isoform of SMYD3 containing Lys at position 13 was used for the biochemical assays, SPR and ITC while the SMYD3 isoform containing Asn at position 13 was utilized for x-ray crystal structures. Protein purification of both isoforms of full length SMYD3 was performed as previously reported [28]. Position 13 of SMYD3 is affected by a naturally occurring SNP, resulting in either an Asn or Lys. Both enzymes were generated and tested (data not shown) to ensure that this amino acid change did not alter compound binding, but only the Asn form was used for crystallography.

C-terminally Avi-tagged SMYD3 (SMYD3-Avi) was cloned into a vector containing an N-terminal His-TEV tag. SMYD3-Avi was produced in *E*. *coli* using IPTG to induce expression at reduced temperatures (16°C) for 16 hours. After harvest by centrifugation, the resulting cell pellets were resuspended, passed through a high pressure homogenizer, and then centrifuged to remove cell debris. The supernatant was passed over a nickel affinity column (Qiagen), washed with buffer containing 20 mM imidazole and SMYD3 was eluted from the column with buffers containing 200 mM imidazole. After dialysis to remove imidazole, the protein was digested by TEV and then passed over an additional nickel affinity column to remove undigested protein and contaminants. The protein was further purified using a Q sepharose column. Biotinylation using His-tagged BirA enzyme occurred after dialysis into a low salt buffer and BirA was removed from the final protein using nickel affinity chromatography. The final purity of the enzyme was >99% as measured using an Agilent Bioanalyzer. Biotinylation was confirmed by mass spectrometry.

Full length FLAG-SMYD2 and C-terminally Avi-tagged full length SMYD2 (SMYD2-Avi) were cloned into pFastbac vectors containing an N-terminal His tag with TEV cleavage site. Virus production was performed using standard protocols and the proteins were coexpressed in either Sf9 (SMYD2) or Hi Five (SMYD2-Avi) cells. After harvest by centrifugation, cell pellets for both proteins were resuspended, sonicated, and then centrifuged to remove cell debris. Protein was purified using nickel affinity chromatography before and after TEV cleavage in a similar fashion as SMYD3. SMYD2 protein was found to be >99% pure as measured by an Agilent Bioanalyzer after the second nickel column. SMYD2-Avi was biotinylated using His-tagged BirA enzyme after dialysis into a low salt buffer and the BirA was removed with a final nickel affinity column. The protein was found to be 99% pure as measured by Bioanalyzer and biotinylation was confirmed by mass spectrometry.

### Biochemical methylation assays

Methyltransferase activity was measured by following the transfer of the tritiated methyl group of ^3^H-S-(5’adenosyl)-L-methionine (SAM, American Radiolabeled Chemicals, Inc.) to a lysine-containing substrate. SMYD3 assays were performed as previously described using N-terminally GST-tagged MAP3K2 protein substrate [28]. For SMYD2, a peptide substrate corresponding to histone H3 residues 1–29 (H3,1–29, ARTKQTARKSTGGKAPRKQLATKAARKSA(K-biotin)-amide) was used. SMYD2 assays were performed in 20 mM bicine, 0.005% bovine skin gelatin, 1 mM TCEP, 0.002% Tween-20, pH 7.5 with 20 nM SAM and 60 nM H3,1–29 peptide in a 50 uL assay volume. Reactions were conducted at room temperature with enzyme and inhibitor incubated for 30 minutes before initiating the reaction with substrates. Dose-response curves were tested in duplicate in each experiment. Reactions were quenched during the linear portion of product formation with a final concentration of 100 µM SAM. Quenched reactions were transferred to a streptavidin-coated flashplate (Perkin Elmer) for capture of the biotinylated peptide. After 2 h incubation at room temperature, the flashplate was washed once with 0.1% Tween-20 and read on a Perkin Elmer Topcount NXT plate reader. Substrate apparent K_M_ values were determined by measuring the initial velocities (*v*_*0*_) in duplicate while varying one substrate at fixed concentration of the second substrate. The resulting data were fit using Eq 1 for peptide substrate and Eq 2 for SAM.

$$v_{0}=\frac{V_{max}S}{K_{M}+S}$$ (1)

$$v_{0}=V_{max}\frac{(E_{T}+S_{T}+K_{M})-\sqrt{{\left(E_{T}+S_{T}+K_{M}\right)}^{2}-4E_{T}S_{T}}}{2E_{T}}$$ (2)

V_max_ is the maximal velocity and K_M_ is the Michaelis constant for the varied substrate S. For the tight-binding treatment of Eq 2, E_T_ and S_T_ are the total concentrations of enzyme and substrate respectively used in the assay.

Percent inhibition by compound (% inh) was calculated from Eq 3

$$\%\;inh=\left(1-(\frac{S_{cmpd}-min}{max-min})\right)\times 100$$ (3)

where S_cmpd_ is the signal in the presence of compound and max and min are the signals for the DMSO and background controls respectively. IC_50_ values were determined from the fitting of percent inhibition data versus inhibitor concentration using Eq 4 where n is the Hill slope.

$$\%\;inh=min+\frac{\left(max-min\right)}{\left(1+{\left(\frac{I}{{IC}_{50}}\right)}^{n}\right)}$$ (4)

Mechanism of inhibition was determined by measuring the inhibitor IC_50_ values while varying the concentration of one substrate at a fixed concentration of the second substrate equal to its K_M_ value. The inhibition constant K_i_ was calculated from fitting the IC_50_ versus varied substrate concentration data using the Cheng-Prusoff equations for mixed-type and noncompetitive inhibition below [31].

Mixed-type:

$${IC}_{50}=\frac{K_{M}+S}{\frac{K_{M}}{K_{i}}+\frac{S}{{\propto K}_{i}}}$$ (5)

Noncompetitive:

$${IC}_{50}=K_{i}$$ (6)

α is the coefficient accounting for differences in inhibitor affinity between binding enzyme forms after versus before substrate binding. Determination of the mechanism of SMYD2 inhibiton with respect to peptide was measured using 1 nM enzyme and 20 nM SAM. Enzyme and substrate concentrations used for the mechanism of EPZ028862 inhibition of SMYD3 are described in S3 Table.

### Methyltransferase enzyme panel

Methyltransferase enzyme screening was done according to general procedures previously described [32].

### Crystallography methods

EPZ033294 was soaked into pre-formed crystals of SMYD2 bound to SAM. SAM was solubilized at 100 mM in DMSO and added to SMYD2 (10 mg/ml in a buffer containing 20 mM Tris, pH 8.0, 100 mM NaCl, and 1 mM TCEP) to a final concentration of 1 mM and incubated on ice for 1 hour. Vapor diffusion methods utilizing hanging drops with a 0.5 mL reservoir were used for crystallization. 1 uL of protein was added to 1 uL of reservoir solution containing 5% v/v ethanol, 100 mM Tris, pH 7.5, and 26% w/v PEG3350. Microseeding was required to produce crystals of SMYD2 of sufficient quality for soaking and subsequent data collection. Crystal trays were incubated at 18°C for 24 hours. EPZ033294 was solubilized at 100mM in DMSO. Crystals were then incubated in a soaking solution containing 5% v/v ethanol, 100 mM Tris, pH 7.5, 26% w/v PEG3350, 4 mM EPZ033294 and 4% DMSO for 24 hours prior to harvesting. Crystals were passed through a cryosolution containing 15% glycerol and 85% soaking buffer prior to freezing in liquid nitrogen.

Crystals of SMYD3 containing an Asn residue at amino acid 13 with EPZ028862 were generated using the soaking protocol previously reported [28].

For SMYD3-EPZ028862, data reduction and scaling were performed using Kylin [33]; for SMYD2-EPZ033294, data reduction and scaling were performed using HKL3000 [34]. Structure determination was performed using previously solved structures of SMYD2 or SMYD3 and visual inspection of electron density maps. Ligand dictionaries were generated using ProDrg [35] within the CCP4 software package [36] and ligand fitting was performed manually. Structure refinement was completed using iterative cycles of refinement and model building using REFMAC [37] and COOT [38], respectively. Data collection and refinement statistics are shown in S2 Table. Structures have been deposited into the Protein Data Bank (SMYD2-EPZ033294 = 5V3H; SMYD3-EPZ028862 = 5V37).

### SPR methods

The SPR binding assay for EPZ033294/SMYD2 was performed at 15°C using the SensiQ system (SensiQ Technoogies, Inc., Oklahoma City, OK). The running buffer contained 25 mM Tris-HCl pH 8.0, 150 mM NaCl, 5 mM DTT, 10 µM ZnCl_2_, 0.05% Tween-20, 10 µM SAM, and 2% DMSO. Avi-tagged biotinylated SMYD2 was immobilized on a neutravidin-coupled BioCap chip up to 6000 RU. Streptavidin on the reference cell was blocked with biocytin. EPZ033294 (0, 3.12, 6.25, 12.5, 25, and 50 µM) was injected for 240 sec at a flow rate of 50 µL/min. Dissociation time was 1000 sec. Analysis of double-referenced data was performed using the 1:1 binding model in QDAT data analysis tool (SensiQ Technologies Inc.).

The SPR binding assay for EZ028862/SMYD3 was performed at 15°C using the Biacore T200 system (GE Healthcare, Marlborough, MA). The running buffer contained 25 mM Tris-HCL pH 8.0, 150 mM NaCl, 1 mM DTT, and 2% DMSO. Avi-tagged biotinylated SMYD3 was immobilized on a streptavidin-coated SA chip up to 1400 RU. The reference cell was blocked with PEG-biotin. EPZ028862 (0, 0.62, 1.9, 5.6, 17, and 50 nM) was injected in the single-cycle kinetics mode at a flow rate of 50 µL/min. Association and dissociation times were 360 and 10,000 sec, respectively. Double-referenced data were analyzed using the 1:1 binding model in BIAevaluation software.

### ITC methods

The ITC binding assay for EPZ033294/SMYD2 was performed using the MicroCal iTC200 system (Malvern Instruments, Malvern UK). The assay buffer contained 20 mM Tris-HCl pH 8.0, 100 mM sodium chloride, 1 mM TCEP, 5% glycerol, 50 μM S-adenosylmethionine (SAM), and 0.1% DMSO. Experiments were performed in three test occasions at 25°C with 200 µM EPZ033294 in the syringe (2 µL injections) and 18 µM of SMYD2 in the cell. The assay background signal was generated by injecting compound into buffer in the absence of enzyme and subtracted from the experimental data. The binding isotherm was fitted using the single site model in the Origin software for data analysis (OriginLab, Northampton, MA).

### Tissue culture and cell lines

Cell lines used in these experiments were obtained from the following sources and were cultured according to conditions specified by the respective cell banks. The following cell lines were obtained from ATCC: Hep3B (HB-8064), SNU-475 (CRL-2236), SNU-423 (CRL-2238), and A549 (CCL-185). KYSE-150 (ACC 375) and KYSE-30 (ACC 351) were purchased from DSMZ and TE4 (RCB2097) was purchased from RIKEN. The cell lines listed above were tested for mycoplasma and were negative. Cells were authenticated by STR method or vendor protocols. All 313 cell lines in the CRIPSR pooled screen were purchased from commercial vendors and were STR authenticated and mycoplasma negative. A broad panel of cancer cell lines was screened in the Eurofins Oncopanel. All cells in this panel were mycoplasma negative and STR verified.

### CRISPR pooled screening

A custom 6.5K sgRNA library, targeting over 600 epigenetic related genes, was ordered from Cellecta, Inc. Cell line screening conditions were previously described for 195 cell lines [39] and this manuscript includes data from these 195 and an additional 118 for a total of 313 cell lines. In brief, cell lines are stably infected with a Cas9 lentiviral vector followed by infected with the custom 6.5K sgRNA lentiviral library. Cell pellets are collected at 14–30 days and sgRNA abundance is assessed via Next Generation Sequencing. Sensitivity was calculated using the Redundant siRNA activity (RSA) score, and is represented here as LogP, as previously described [39].

### Individual sgRNA CRISPR infections

Single expression system lentivirus containing Cas9 and sgRNA for all targets were purchased from Cellecta, Inc. The sequences for the sgRNAs are as follows: SMYD3 sgRNA—CGTCGCCAAATACTGTAGTG, EZH2 sgRNA—TTGCGGGTTGCATCCACCAC, and HBE1 sgRNA—CTTCCACATTCATCTTGCTC. On day 0, cells were plated at a density of 1800 cells/cm^2^ in a 100mm culture dish containing 10mL complete medium. 24h post plating the cells were infected with sgRNAs at MOI3 in the presence of 4ug/mL Polybrene (Millipore, #TR-1003-G). Viral media was removed 24h post infection and selection by puromycin (1ug/mL) was initiated 48h post infection. Infected cells were cultured under puromycin selection for 30 days.

### MAP3K2 cellular methylation assay

The protocols for the in cell western assay for SMYD3-MAP3K2 were previously reported in Mitchell *et al* [28]

### Western blotting

Cells were lysed in 1X RIPA Buffer containing 10% SDS and Halt™ Protease Inhibitor Cocktail (Thermo Fisher Scientific) and incubated on ice for 30 minutes before sonication (Amplitude 20%/10sec) twice. Lysates were spun at 13.2 rpm at 4°C for 10min and normalized for protein concentration by BCA assay (Thermo Fisher Scientific). Lysate was fractionated on a 4–12% Bis-Tris Protein gel (Thermo Fisher Scientific) and transferred using the iBlot (Program 3–8 minutes, Nitrocellulose transfer stacks). Blots were imaged using the Odyssey Imaging System (LICOR Biosciences). The blots were probed with the following 1^o^ antibodies in Odyssey Blocking Buffer (LI-COR Biosciences): rabbit anti-SMYD3 Antibody (Thermo Fisher Scientific, # PA5-31919, 1:1,000 dilution), mouse anti-GAPDH (Millipore, #CB1001, 1:10,000 dilution), and rabbit anti-BTF3 (Abcam, ab66940, 1:50 dilution). BTF3me1 is a custom generated polyclonal antibody, affinity purified from serum of rabbits injected with adjuvant conjugated peptide corresponding to methylated K1 of BTF3 (K(Me)-ETIMNQEKLAKC). Antibody was validated by western blot on lysates derived from 293T cells over-expressing SMYD2 and KYSE-150 cells treated with LLY-507. (S7 Fig). Membranes were probed with Alexa Fluor® 680 Donkey anti-rabbit IgG (Thermo Fisher Scientific, #A10043, 1:20,000 dilution) and IRDye 800CW Donkey anti-Mouse IgG (LI-COR Biosciences, #926–32212, 1:20,000 dilution) secondary antibodies.

### Cellular thermal shift assay

Cellular thermal shift assays were performed at Pelago Bioscience (https://www.pelagobio.com)

#### Generation of melt and shift curves of SMYD3

Equal volumes live A549 cells in HBSS and 2x compound concentration in HBSS were mixed, resulting in a final cell concentration of 20 million cells/mL and 10 μM compound. An incubation with 0.1%DMSO only was prepared in parallel as negative control. Incubations were performed during 60minutes at 37°C with continuous mixing. The treated cells were divided into 50 μl aliquots and subjected to a 12-step heat challenge between37 and 63°C for 3 minutes. The heat step was followed by immediate lysis by three rounds of freeze thawing in liquid nitrogen followed by centrifugation at 20000 x g for 20 minutes to pellet precipitated protein. 30 μl of the supernatants were mixed with 15 μl gel loading buffer (NuPAGE LDS sample buffer, Life Technologies) and 10 μl of each mixture was loaded per lane on gels. Protein amounts were detected using Western Blot techniques as described above for SMYD3.

#### Generation of concentration response curves of SMYD3

Live A549 cells in HBSS were divided into 25 μl aliquots and an equal volume of HBSS containing 2xthe incubation compound concentration was added. 7 step dilution concentration response series of the compounds in 1% DMSO were applied together with 1% DMSO only as control. The concentration series ranged from 100 μM to 10 nM. The final cell concentration was 20 million cells/mL and compound incubation was performed during 60 minutes at 37°C with continuous mixing. The treated cells were subjected to a heat challenge at 47°C (as determined from the melt curves) for3 minutes, followed by lysis by three rounds of freeze-thawing and separation of precipitated proteins by centrifugation at 20000g for 20 minutes. 30 μl of the supernatants (soluble fraction) were mixed with 15 μl gel loading buffer (NuPAGE LDS sample buffer, Life Technologies) and 10 μl of each mixture was loaded per lane on a gel. Protein amounts were detected using Western Blot techniques as described above for SMYD3.

## Results

### SMYD2 small molecule inhibition

**Fig 1:**
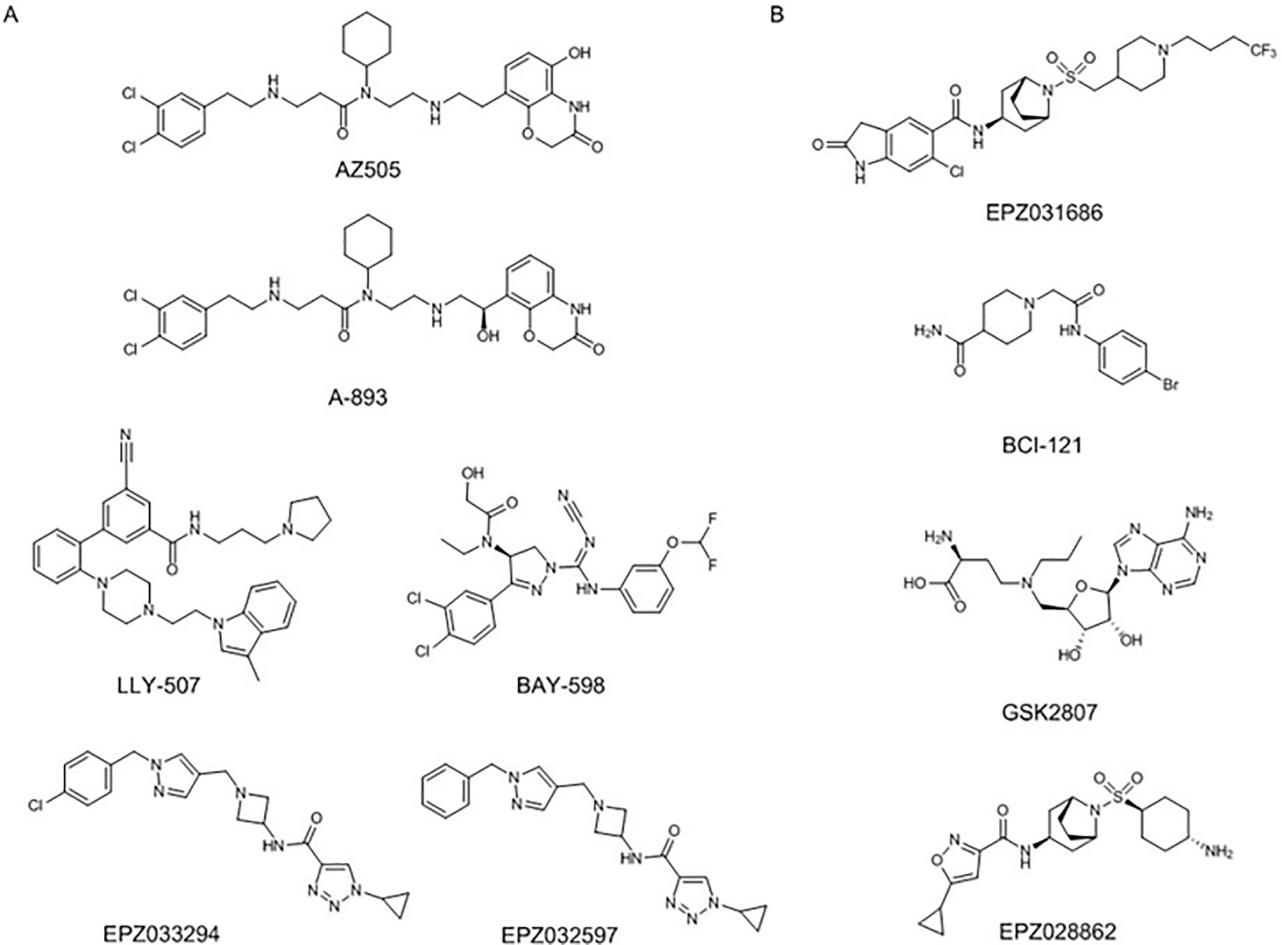
**Chemical structures of SMYD2 (A) and SMYD3 (B) inhibitors**.

**Fig 2:**
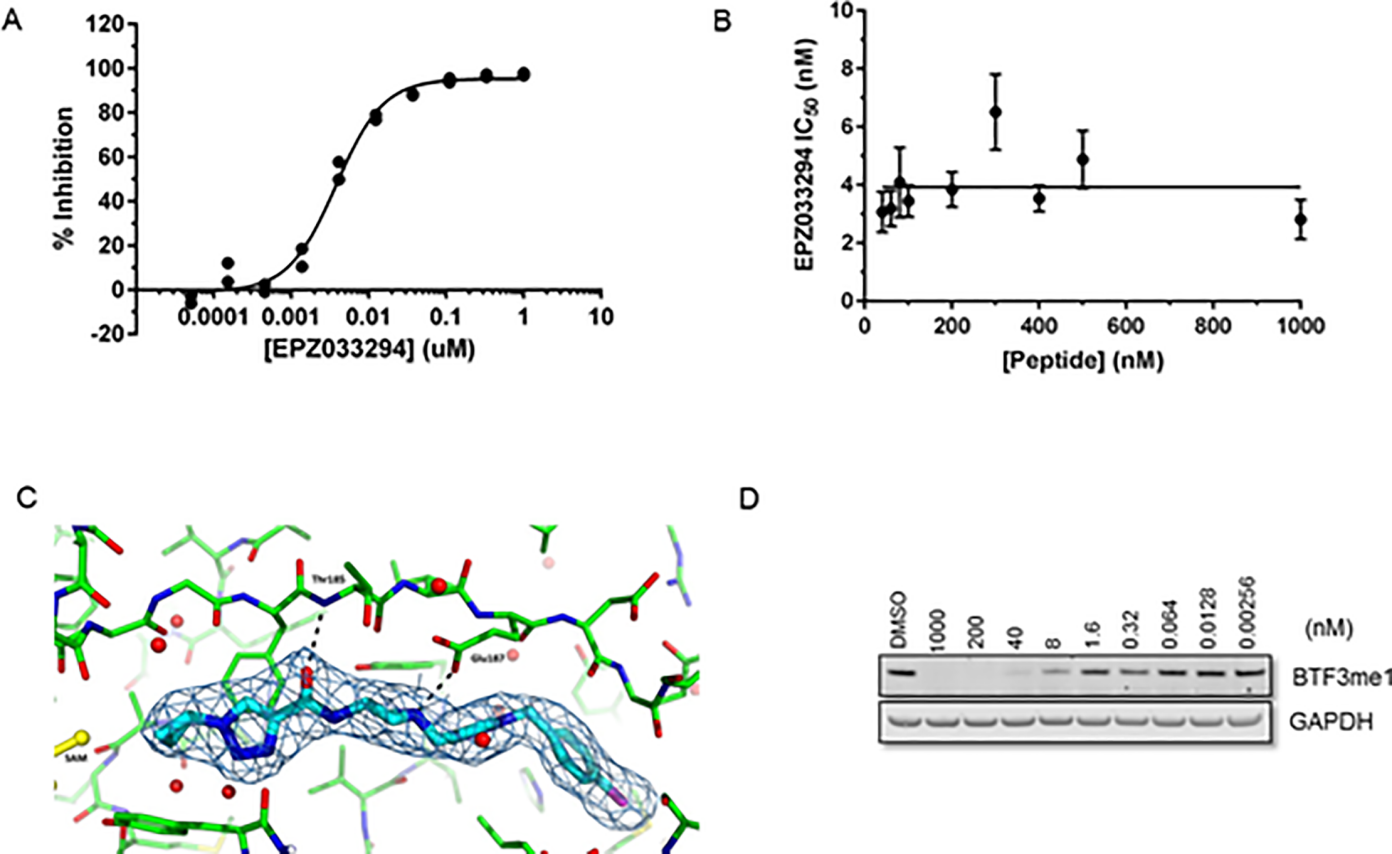
Characterization of EPZ033294 as an inhibitor of SMYD2. A) Representative SMYD2 biochemical dose-response curve for EPZ033294. IC_50_ value and standard deviation of 3.9 ± 0.3 nM was determined from 2 independent experiments. B) EPZ033294 IC_50_ values as a function of peptide concentration illustrating noncompetitive inhibition. IC_50_ values with their standard error and the fit line calculated from a single experiment. C) Structure of EPZ033294 (cyan) with SMYD2 (green) and SAM (yellow) (PDB ID 5V3H). Electron density (2Fo−Fc, 1σ) for the compound is shown. Hydrogen bonds are indicated as dashed lines. D) Western blot of BTF3 methylation showing dose dependent effects of EPZ033294 and a cell biochemical IC_50_ of 2.9 nM. Data shown is representative of three independent experiments.

Fig 1A illustrates the chemical structures of SMYD2 inhibitors that have been reported previously and of EPZ033294 and EPZ032597, new inhibitors representing a completely novel pharmacophore series. The biochemical, cellular, and physicochemical properties of these various inhibitors are summarized in Table 1. EPZ033294 is an inhibitor of SMYD2 that was optimized from an initial hit from Epizyme’s proprietary histone methyltransferase-biased library using a radiometric assay with tritiated SAM and an H3 peptide as substrates. EPZ033294 inhibits the enzymatic activity of SMYD2 with a potency of 3.9 nM (Fig 2A) and is noncompetitive with respect to peptide substrate (Fig 2B) and either noncompetitive or uncompetitive with respect to SAM. SAM displays a high affinity for SMYD2 (Supporting Information S1 File.) so a dual titration of inhibitor and SAM could not be performed to determine the mechanism of inhibition with respect to SAM. Instead, SAM dependence was tested by measuring IC_50_ values at two SAM concentrations differing by 10-fold; the resulting IC_50_ values were similar (Supporting Information S1 File.), inconsistent with a competitive mechanism. In addition, EPZ033294 and EPZ032597 were tested against a panel of 15 additional methyltransferase enzymes to check the selectivity of the compounds for SMYD2. For all enzymes tested, including the closely related enzyme SMYD3, no inhibition was seen up to 10 μM (S1 Table).

**Table 1:** Biochemical and cellular potencies and physicochemical properties of SMYD2 and SMYD3 inhibitors used in this study.

Target	Compound	Biochemical IC_50_ (nM)	Intracellular Methylation IC_50_ (µM)	Cellular Proliferation IC_50_ (µM)	M.W. (Da)	Total P.S.A. (Å^2^)	cLogP
**SMYD2**	AZ505	51 (3)^1^	13.900 (1)^1^	12.8 (2)^1^	577.55	102.9	5.22
	LLY-507	17 (3)^1^	0.691 (3)^1^	1.8 (3)^1^	574.77	65.9	6.86
	BAY-598	27 ^2^	0.058 ^2^	>20 ^2^	525.34	113.6	5.85
	EPZ032597	16 (2)^1^	0.031 (2)^1^	>40 (3)^1^	377.44	80.9	1.73
	EPZ033294	3.9 (2) ^1^	0.0029 (3) ^1^	>40 (2)^1^	411.89	80.9	2.33
**SMYD3**	GSK2807	130 ^3^					
	BCI-121	11,800 ^4^		>100 ^4^			
	EPZ030456	4.0 ^5^	0.051 ^5^		572.12	110.9	1.38
	EPZ031686	3.0 ^5^	0.036 ^5^		591.09	100.0	2.18
	EPZ028862	1.8 (2)^1^	0.032 (3)^1^	>40 (2) ^1^	422.54	118.5	0.31

^1^ Mean value from this study. Value in parenthesis indicates number of experimental determinations. Reported proliferation IC_50_s were measured in KYSE-150 (SMYD2) or HepG2 (SMYD3).

^2^ Data from Eggert *et al* (2016).

^3^ Data from Van Aller *et al* (2016)

^4^ Data from Peserico *et al* (2015)

^5^ Data from Mitchell *et al* (2016)

The binding mode suggested by enzyme kinetics is consistent with the crystal structure of the ternary SAM-SMYD2- EPZ033294 complex (Fig 2C). The cyclopropyl triazole moiety occupies the lysine channel with the amide carboxyl group hydrogen-bonding to the backbone amide NH of Thr185 and the azetidine nitrogen interacting with the side chain of Glu187 (Fig 2C); both amino acids are also engaged in hydrogen bonding interactions with peptide substrates [13]. Compared to all other published SMYD2 inhibitors, EPZ033294 binds in a distinct and unique manner, traversing the peptide binding site and inducing a pocket into which the hydrophobic tail of the molecule is inserted (S1 Fig).

SMYD2 catalyzes the methylation of lysine residues on several proteins, including BTF3 [40]. Monomethylation of BTF3 (BTF3me1) by SMYD2 proved to be a convenient and reliable measure of intracellular SMYD2 activity and inhibition. Western blot analysis of intracellular levels of BTF3me1 as a function of EPZ033294 concentration demonstrated a concentration-dependent inhibition of this mark with an IC_50_ of 2.9 nM (Fig 2D, Table 1). Despite relatively potent inhibition of SMYD2 biochemical activity (Table 1), the previously reported compounds AZ505 and LLY-507 demonstrated inhibition of intracellular BTF3me1 (Table 1), and were significantly less potent in this assay than EPZ033294 or the reported cellular methylation activity of BAY-598 [10] (Table 1).

**Fig 3:**
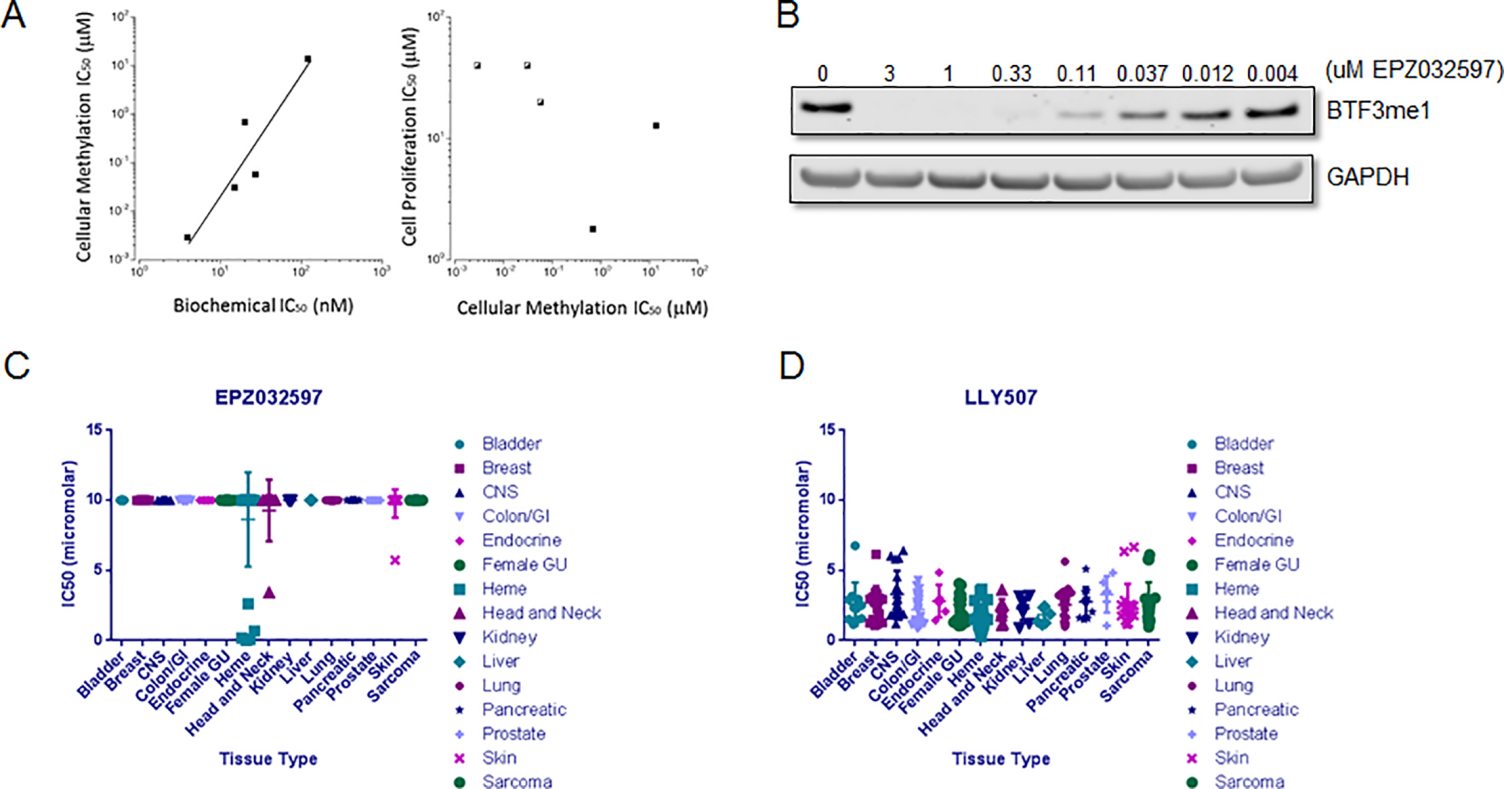
Anti-proliferative activity of SMYD2 inhibitors. (A) Correlation plots of (left) cellular methylation IC_50_ as a function of biochemical IC_50_ and (right) cell proliferation IC_50_ as a function of cellular methylation IC_50_ for SMYD2 inhibitors. (B) Western blot of BTF3 methylation showing dose dependent effects of EPZ032597. Data is representative of two independent experiments. (C) The effect of EPZ032597 on proliferation in a broad panel of cancer cell lines. (D) The effect LLY507 on proliferation of a broad panel of cancer cell lines. Values for C) and D) are the average of three biological replicates; error bars represent standard deviations (not readily visible on scale for all points). The 10 μM value represents the highest dose tested.

Thus, 5 compounds representing 4 distinct pharmacophore series of SMYD2 inhibitors are now available for testing the dependence of cancer lines on SMYD2 activity. We hence measured the ability of these compounds to affect the growth of the esophageal cancer cell line KYSE-150, a SMYD2 over-expressing cell line previously reported to require SMYD2 activity for proliferation (Fig 1 and Table 1). As illustrated in Fig 3A, all 5 SMYD2 inhibitors demonstrated a good correlation between their potency for inhibiting the enzyme in cell-free assays and for inhibiting intracellular BTF3 methylation. In stark contrast, however, no correlation is seen between the potency for BTF3 methylation inhibition and anti-proliferative activity among these compounds (Fig 3B).

We next tested proliferation in response to EPZ032597, a compound similar to EPZ033294 in structure with a cellular IC_50_ of 31 nM (Fig 3B, Table 1), for effects against a panel of 240 cell lines (Eurofins Panlabs Oncopanel) representing a broad variety of cancer cell lines of differing origin (see S6 Table for more information). EPZ032597 was inactive as an anti-proliferative agent in nearly all tested cell types with a proliferative IC_50_ not observed at concentrations up to 10 μM in a 10-day assay (Fig 3C). We note that the data illustrated in Fig 3D represents the results of an initial screen of 240 cancer cell lines and in this initial screen several hematologic cancer cells appeared to display lower anti-proliferative IC_50_ values for this compound. Repeat testing of these cell lines demonstrated that the lower IC_50_ values represented modest growth inhibition (<50%) without a strong concentration dependency. These findings are similar to results using BAY-598, an inhibitor with a different mechanism of inhibition [10]. In contrast, LLY-507 displayed anti-proliferative activity against cancer cell lines of all types tested (Fig 3D) irrespective of SMYD2 expression (S2 Fig), with the majority of cells demonstrating anti-proliferative IC_50_ values between 1 and 5 µM. Based on the aggregate data presented here, in Eggert *et al* [10], and from the additional screening data now available (http://www.chemicalprobes.org; http://www.thesgc.org/chemical-probes), we conclude that the anti-proliferative activity observed with LLY-507 likely results from off-target activities of this compound and not from the specific inhibition of SMYD2 enzymatic activity.

### SMYD3 small molecule inhibition

**Fig 4:**
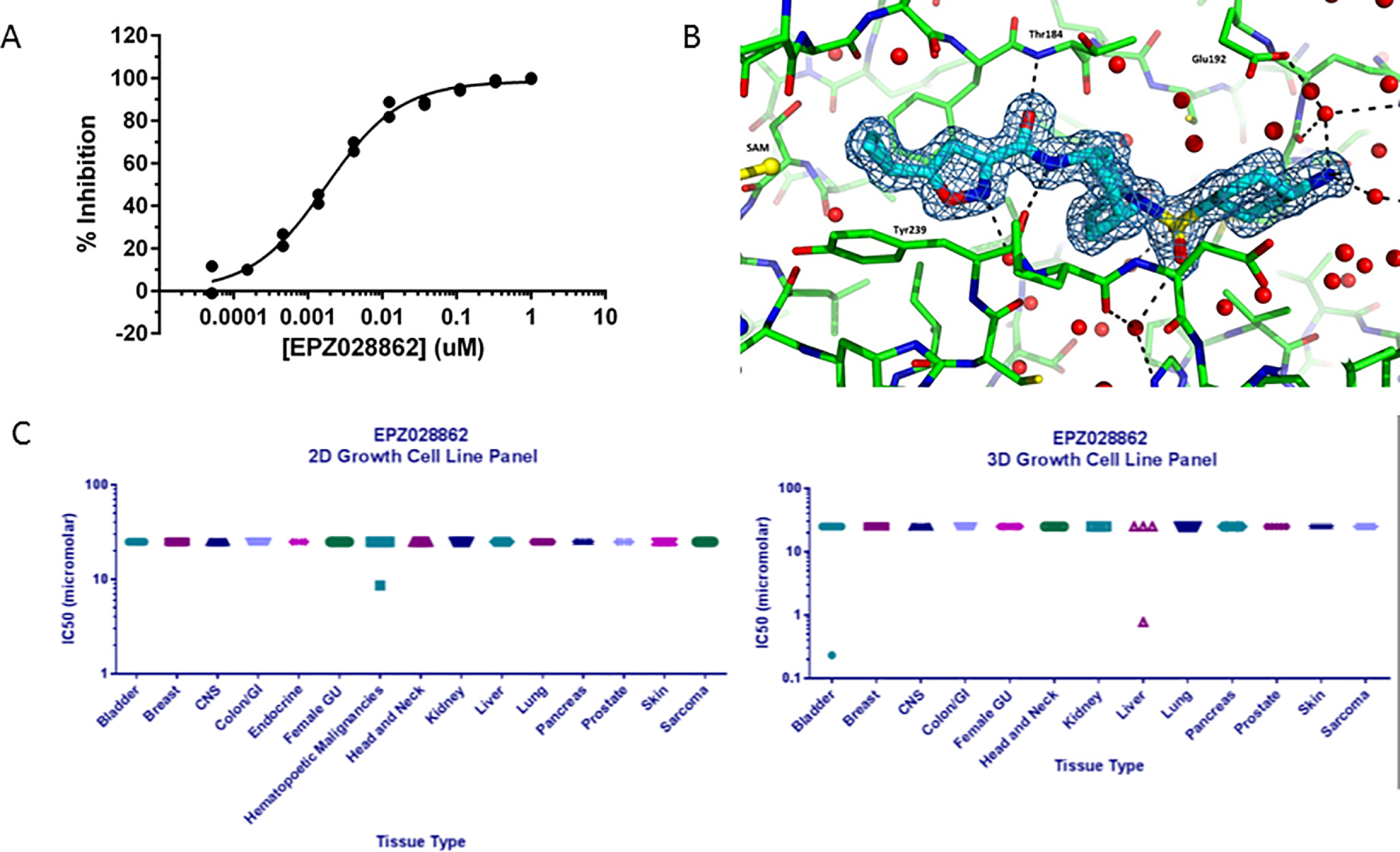
Characterization of EPZ028862 as an inhibitor of SMYD3. A) Representative SMYD3 biochemical dose-response curve for EPZ028862 with a mean IC_50_ value and standard deviation of 1.80 ± 0.06 nM from 2 experiments. B) Structure of EPZ028862 (cyan) with SMYD3 (green) and SAM (yellow) (PDB ID 5V37); water molecules are represented with red spheres. Electron density (2Fo−Fc, 1σ) for the compound is shown. Hydrogen bonds are indicated as dashed lines. C) Anti-proliferative activity of the SMYD3 inhibitor EPZ028862 across a broad panel of cancer cell lines in 2D culture (left) and in 3D culture (right). The 25 μM value represents the highest dose tested. Each value represents the mean of three replicates. Error bars represent the standard deviation (not readily visible on scale).

Few small molecule inhibitors of SMYD3 have been reported, and the chemical structures of these are illustrated in Fig 1B. The SAM mimetic GSK2807 [30] is a 130 nM inhibitor of the SMYD3 enzyme in cell-free biochemical assays, but no cellular data was reported for this compound. BCI-121 [29] was identified as a SMYD3 inhibitor through virtual docking experiments and was subsequently shown to bind to SMYD3 with a K_d_ of 11.8 µM. This compound was shown to inhibit proliferation of several cell lines by 20–30% at 100 µM. The oxindole sulfonamides and sulfamides, EPZ030456 and EPZ031686 [28], show low nanomolar inhibition of SMYD3 enzymatic activity and intracellular concentration-dependent inhibition of methylation of the SMYD3 substrate MAP3K2, originally identified by Mazur *et al*. [21]. Structure-activity relationship studies of SMYD3 inhibitors led to the isoxazole sulfonamide series exemplified by EPZ028862, another molecule displaying similarly potency against SMYD3 in biochemical and cellular assays with physicochemical properties suitable for *in vivo* studies (Fig 4A and 4B; Table 1 and S2 Table). In addition, EPZ028862 was tested against a panel of 15 methyltransferase enzymes to check the selectivity of the compound for SMYD3. For all enzymes tested, including the closely related enzyme SMYD2, no inhibition was seen up to 10 μM (S1 Table).

EPZ028862 was therefore used as a tool compound to further probe SMYD3 biology. However, unlike SMYD2, cellular target engagement for SMYD3 could not be determined by endogenous substrate methylation; methylation of the reported SMYD3 substrates (H4K5 or MAP3K2) [21, 22] were not readily detected in any tissue culture cell line tested by western blotting (data not shown). Instead, cell activity of SMYD3 was determined using a previously documented engineered system [28] in which both SMYD3 and MAP3K2 were cotransfected into 293T cells and inhibition of Lys260 methylation of MAP3K2 was measured. Therefore, in order to provide support that EPZ028862 binds endogenous SMYD3 rather than only the overexpressed protein, a cellular thermal shift assay (CETSA) was performed that reported a 1.4 μM EC_50_ for SMYD3 binding (S3 Fig) and verified target engagement of EPZ028862 for endogenous SMYD3. It is important to note that the EC_50_ measured by CETSA represents the binding affinity of EPZ028862 to SMYD3 at 47 degrees and likely underestimates the potency at the physiologically relevant temperature of 37 degrees.

With verification of inhibition of SMYD3 in cells, we then specifically tested how inhibition of SMYD3 by EPZ028862 affected cell proliferation *in vitro*. We studied the effects of EPZ028862 in a panel of KRAS mutant lung cancer cell lines including A549, for which RNAi-based knockdown studies had previously suggested an important role for SMYD3 in proliferation [21]. Treatment of NSCLC and other lung cancer cell lines with and without KRAS mutations at concentrations as high as 25 µM had no impact on proliferation (S5 Table). Additionally, a previous report indicated that SMYD3 ablation by RNAi enhanced the effect of the MEK1 inhibtor, trametinib. Mazur *et al* [21] However, the effects of trametinib were unaffected by combination with 1 μM EPZ028862 (S4 Fig). We subsequently extended these studies to a broad panel of 240 cancer cell lines (Eurofins Panlabs Oncopanel) across a variety of indications (Fig 4C, left panel); again, no proliferative IC_50_ was observed for this compound in any of the cancer cell lines at concentrations as high as 25 µM. Additionally, to explore reports suggesting that SMYD3 plays a role in three-dimensional growth [14, 22], a panel of 140 cell lines grown in matrigel were treated with EPZ028862; again, no growth effects were observed up to 25 µM (Fig 4C, right panel).

### Genetic ablation of SMYD2 or SMYD3

**Fig 5:**
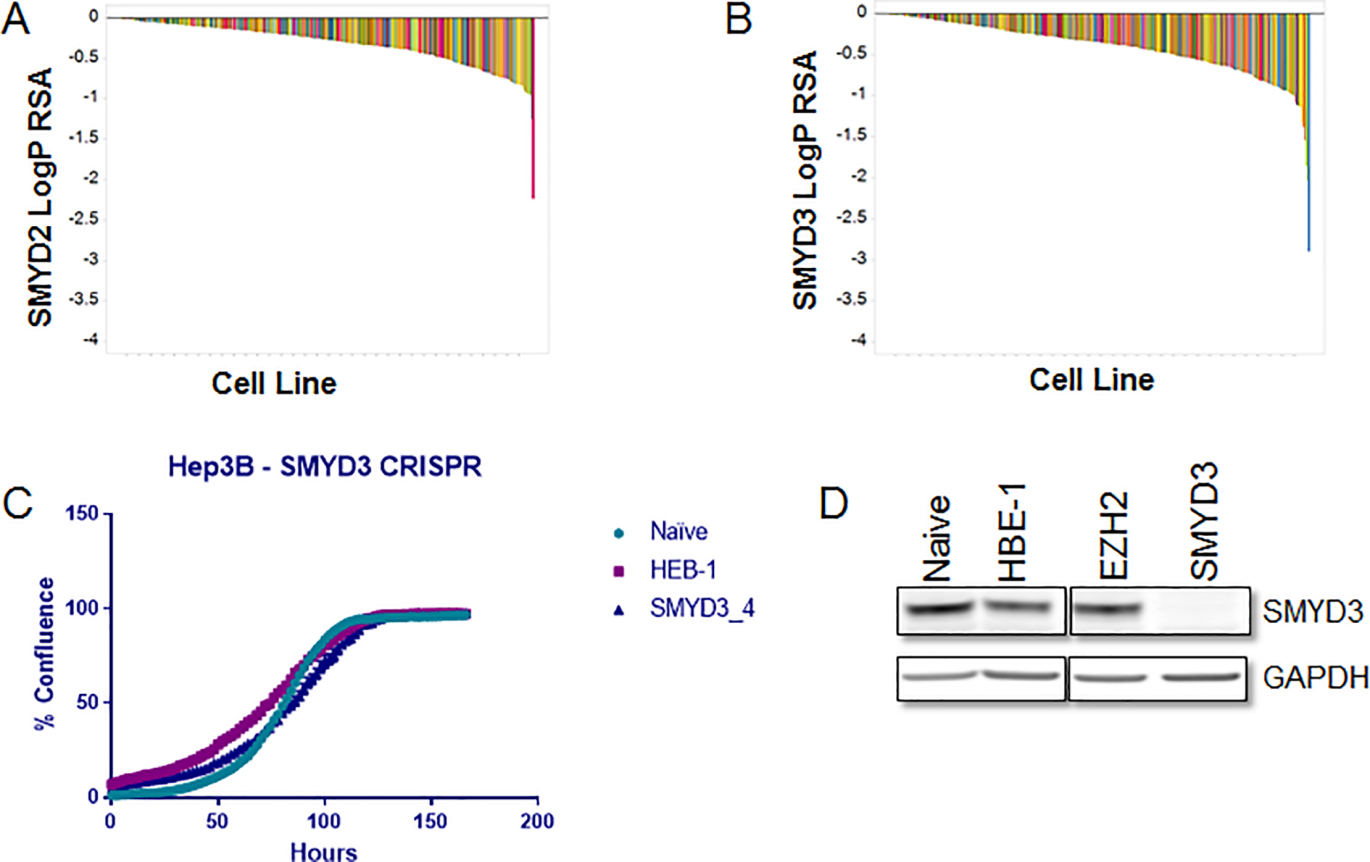
Gene ablation techniques show no dependence on SMYD2 or SMYD3 for cancer cell proliferation. Waterfall plot representing LogP RSA scores for sgRNAs targeting A) SMYD2 and B) SMYD3. 313 cell lines were infected with a library of 6500 sgRNAs targeting 600 different genes. LogP RSA scores represent depletion of guides from an infected cell population. Each bar represents a different cell line. Bars are colored by cancer subtype. C) Percent confluency of Hep3B cells infected with CRISPR viruses containing CAS9 and sgRNAs targeting HBE-1, EZH2 (negative controls) or SMYD3. Cell density was evaluated using an Incucyte Zoom. Growth curves were initiated 24 days following virus infection and puromycin selection. Plotted data is the average of three biological replicates. Error bars represent standard deviation (not readily visible on scale). D) SMYD3 western blot of lysates derived from Hep3B cells infected with CAS9 and SMYD3 sgRNA. Parental Hep3Bs and Hep3Bs stably infected with HBE-1, EZH2 (negative controls) or SMYD3 were lysed and probed for SMYD3 levels by western. GAPDH levels were evaluated as a loading control.

CRISPR/Cas9 pooled screening has potential advantages over RNAi-based methods as it may have fewer off-target effects and leads to gene knockout as opposed to only gene knockdown. This may be especially important for epigenetic targets, which are likely to require almost complete target inhibition to observe the phenotype. We designed a custom CRISPR lentiviral library of 6500 small guide RNAs (sgRNAs) targeting 640 epigenetic genes [39] and screened 313 cancer cell lines for proliferation effects. These 313 cancer cell lines cover a variety of solid tumor indications (S6 Table for more information). Importantly, the screen contained both positive and negative controls, including the pan-essential genes PLK1 and EIF4A3 (which shows depletion in nearly all cell lines tested: S5 Fig) and 60 non-targeting negative control sgRNAs which induce no proliferation phenotype [39]. Both SMYD2 and SMYD3 were included in this library; however, none of the cell lines tested showed any robust proliferation effect upon loss of either enzyme (Fig 5A and 5B). Included in the cell lines tested by CRISPR are a panel of 26 esophageal cell lines, including KYSE-150, which has been reported to be sensitive to SMYD2 inhibition [11] and a panel of 18 KRAS mutant NSCLC cell lines that include the A549 cell line that has been reported to be sensitive to SMYD3 knockdown [21]. Subsequent experiments employing individual sgRNAs in three hepatocellular carcinoma cell lines reported to be dependent on SMYD3 activity [15, 22] showed little response to ablation of protein expression. Hep3B cells were infected with lentivirus containing Cas9 and an sgRNA targeting Exon 2 of SMYD3. Following 30 days of selection, SMYD3 protein detection and growth rates of infected cells were determined. Hep3B cells infected with SMYD3 sgRNA virus have undetectable levels of SMYD3 but showed similar growth characteristics to controls indicating that loss of this enzyme is well tolerated by this cell line (Fig 5C and 5D). Growth curves at a 12-day time point following infection of Hep3B cells also showed little difference in growth between controls and SMYD3 targeting sgRNAs (data not shown). Similar results were also obtained for two additional HCC cell lines, SNU-475 and SNU-423 which had both been previously reported to be sensitive to SMYD3 ablation [15, 22](S6 Fig). The findings that SMYD2 and SMYD3 targeted CRISPR-Cas9 knockout are well tolerated in every cell line tested and inhibitors of SMYD2 and SMYD3 have almost no detectable effects on cell proliferation, we conclude that neither the activity nor expression of these enzymes is required for *in vitro* cancer cell proliferation.

## Discussion

The data presented in this work offer strong evidence that in contrast to some of the literature, SMYD2 and SMYD3 are not required for cancer proliferation in vitro. The lack of proliferation activity of SMYD2 and SMYD3 inhibitors or SMYD2 and SMYD3 CRISPR knockout in any tested cell line, including many of those reported to be sensitive to SMYD2 or SMYD3 RNAi in the literature may suggest that the high expression level of SMYD2 and SMYD3 observed in a wide variety of cancers has another function than simply regulating cell growth. A number of sophisticated *in vivo* studies on SMYD2 and SMYD3 using knockout mice have demonstrated that these enzymes play a key role in oncogenesis [8, 9, 21, 27]. Mazur *et al*. demonstrate that SMYD3 deficiency reduces tumorigenesis induced by mutant KRAS in both pancreas and lung [21]. Consistent with these findings, Sarris *et al*. show that SMYD3 is required for chemically induced liver and colon carcinogenesis [27]. A similar model for SMYD2 has revealed that this enzyme also plays a role in KRAS driven pancreatic cancer initiation and progression and is important for the cellular response to genotoxic agents (e.g. gemcitabine) [8]. SMYD2 was also shown to be required for MLL-AF9-induced leukemogenesis in vivo [9]. Based on these *in vivo* studies and the absence of any effects observed in cell culture, it is possible that SMYD2 and SMYD3 play a role in oncogenesis that that may be more easily uncovered by *in vivo* studies. These enzymes may be vital to an early initiation step of oncogenesis or may play a role that effects cell growth only *in vivo*, like the regulation of the tumor microenvironment, angiogenesis or immune evasion. Recent reports may also indicate a role for SMYD2 and SMYD3 in the systemic response to cancer. For instance, SMYD3 has been implicated in the differentiation of T regulatory cells, which regulate immune checkpoints in cancer [41, 42]. SMYD2 has been shown to suppress the activation of macrophages, which have also been implicated in the immune response to cancer [41]. The work presented here indicate that despite many publications suggesting a role for SMYD2 and SMYD3 in autonomous proliferation or survival, the mechanism by which SMYD2 and SMYD3 might regulate tumorigenesis remains largely unknown outside of the publications who have explored these enzymes *in vivo* [8, 21, 27, 42]. It now remains to be seen whether chemical inhibition of SMYD2 or SMYD3 *in vivo* would affect an already existing tumor and hence be a valuable drug target.

Workman and Collins[43] noted that evolution of probe molecules for a specific target can result in refinement of hypotheses regarding their biological effects. The availability of high-quality probe compounds against these two targets creates the opportunity for the further exploration of the biology and pathobiology of SMYD2 and SMYD3. The work presented here, along with the mouse genetic studies suggest that these two enzymes may play a complex role in cancer biology that could be further studied using high quality chemical matter.

Determining the proper use of genetic tools in identification of novel cancer targets has been a continuing process since the discovery of gene knockout and RNAi technologies. Although estimates have been attempted [44, 45], it is unknown how much of the literature in cancer biology will ultimately be effected by undiscovered artifacts associated with RNAi technology. The introduction of CRISPR pooled screening has fundamentally changed how genetic ablation experiments are performed and the results interpreted. For example, a recent study employing CRISPR pooled screening showed that the maternal embryonic leucine zipper kinase (MELK) is not involved in cancer cell line proliferation, in stark contrast to a large number of previous studies [46]. Like MELK, the proposed roles of SMYD2 and SMYD3 for *in vitro* cell proliferation have also been disproven with CRISPR. The invalidation of the published roles for MELK, SMYD2 and SMYD3 suggest that a much higher bar must be set for the use of genetic tools in cancer biology.

In summary, the work described in this manuscript provides extensive evidence that SMYD2 and SMYD3 are not required for *in vitro* cell line proliferation, and that determining what role SMYD2 or SMYD3 might play in oncology will require further studies. Moreover, we feel that the inhibitors described here will provide valuable research tools for studying these enzymes further.

## Supporting information

S1 File Supplemental materials and methods, compound synthesis, biochemical characterization and ADME properties of SMYD2 and SMYD3 inhibitors. (PDF)

S1 Fig Crystal structure of EPZ033294 bound to SMYD2. (A) Two molecules of EPZ033294 (molecule 1 = cyan; molecule 2 = purple) were seen in the peptide binding site of SMYD2 (green). SAM (yellow) is show in stick representation. (B) The tail of EPZ033294 molecule 1(green) induces a hydrophobic pocket when compared to structures of SMYD2 (grey; PDB 3TG4 [47]); SMYD2-ERα peptide (yellow; PDB 4O6F [48]) and SMYD2-p53 peptide (blue; PDB 3S7F [13]). (C) EPZ033294 (green) has a unique binding mode compared to known SMYD2 inhibitors BAY598 (cyan; PDB 5ARG [10]), A893 (magenta; PDB 4YND [12]), LLY507 (yellow; PDB 4WUY [11]), and AZ505 (orange; PDB 3S7B[13]).
(PDF)

S2 Fig Scatter plot showing mRNA expression of SMYD2 does not correlate with the IC_50_ value of LLY507. (PDF)

S3 Fig CETSA with EPZ028862 confirms cellular target engagement. A) Representative western blot showing thermal stability of SMYD3 with and without 100 micromolar EPZ028862. Largest thermal shift with and without EPZ028862 was observed at 47 degrees C. B) Dose-response SMYD3 CETSA for EPZ028862. CETSA EC50 of EPZ028862 at 47 degrees is approximately 1.4 μM. (Representative of 3 western blots).
(PDF)

S4 Fig SMYD3 inhibitor treatment does not affect IC_50_ of trametinib. A549 cells were treated with varying concentrations of trametinib alone (left) or in combination with 1 µM EPZ028862(right) for 2, 5 and 7 days. Addition of EPZ028862 has no effect on growth inhibition by trametinib in A549 cells. Plotted data is the average of three biological replicates. Error bars represent standard deviation.
(PDF)

S5 Fig **CRISPR pooled screen data for 313 cell lines for two pan-essential controls, PLK1 (A) and EIF4A3 (B).** On the y-axis is the sensitivity p-value.
(PDF)

S6 Fig Growth of SNU-475 and SNU-423 cell lines were evaluated following SMYD3 knockout. A and C show Incucyte growth curves of both cell lines with virus containing a sgRNA targeting the fetal hemoglobin gene (HBE1) or exon 2 of SMYD3. Plotted data is the average of three biological replicates. Error bars represent standard deviation. B and D confirm persistent knockout of SMYD3 in SMYD3 sgRNA infected cells out to 19 days.
(PDF)

S7 Fig Development of BTF3K1me1 antibody. Serum from rabbits injected with adjuvant conjugated peptide corresponding to methylated K1 of BTF3 (K(Me)-ETIMNQEKLAKC) was tested for activity by western blot. Lysates from 293T cells overexpressing SMYD2 or KYSE-150 cells treated with increasing concentrations of LLY-507 were collected. Western blot analysis was performed using affinity purified anti-BTF3me1 antibody. Cells over-expressing SMYD2 show an increase in anti-BTF3me1 signal. Cells treated with LLY-507 show a decrease in anti-BTF3me1 signal.
(PDF)

S8 Fig SMYD2 substrate steady-state kinetics. Initial velocities with their standard error from timecourse data in duplicate are shown as function of substrate concentration. Rates for varied peptide at 2 nM SMYD2 and 50 nM SAM were fit using eq 1 which gives a K_M_ value for H3,1–29 of 66 ± 11 nM from 1 experiment (A). Rates for varied SAM at 1 nM SMYD2 and 60 nM H3,1–29 were fit using Eq 2 which gives a K_M_ value for SAM of 0.34 ± 0.07 nM from 1 experiment (B).
(PDF)

S9 Fig Mechanism of inhibition of SMYD2 by EPZ032597. IC_50_ values with their standard error from Eq 4 are plotted as a function of peptide concentration. EPZ032597 inhibition is best described as noncompetitive versus peptide using Eq 6 with a K_i_ value of 21.5 ± 1.5 nM from one experiment.
(PDF)

S10 Fig Biophysical characterization of EPZ033294 to SMYD2. (A) One representative thermogram for ITC binding of EPZ033294 to SMYD2 is shown. Stoichiometry of binding in this experiment was found to be 0.7. (B) Measurement of binding of EPZ033294 to SMYD2 by SPR assay. The dissociation constant (K_D_) was determined to be 5 nM, with a *k*_on_ = 5 x 10^5^ M^-1^s^-1^ and *k*_off_ = 0.003 s^-1^.
(PDF)

S11 Fig Mechanism of inhibition of SMYD3 by EPZ028862. EPZ028862 IC_50_ values with their standard error from Eq 4 are plotted as a function of MEKK2 (A) and SAM (B) concentration using the filterplate assay. EPZ028862 inhibition is best described as noncompetitive versus MEKK2 (Eq 6) and mixed-type inhibition versus SAM (Eq 5). Values for the inhibition constants are shown in S3 Table.
(PDF)

S12 Fig Binding of EPZ028862 to SMYD3 by SPR. A single representative sensogram (red) is shown with the calculated fit (black). Kinetic constants *k*_on_ (6.1 ± 1.1 x 10^5^ M^-1^s^-1^) and *k*_off_, (2.1 ± 0.57 x 10^−4^ s^-1^) were based on fitted values from seven replicate measurements (mean ± standard deviation).
(PDF)

S13 Fig Superposition of EPZ028862 (cyan) and EPZ030456 (grey; PDB 5CCM [28]) shows the overall binding mode of the compounds in the SMYD3 (green) binding site is similar despite the different groups bound in the lysine channel and differing tail moieties. SAM (yellow) is shown in stick representation.
(PDF)

S1 Table Protein methylatransferase selectivity panel. All reported results for off-target enzymes were tested in duplicate.
(PDF)

S2 Table Crystallographic data collection and refinement statistics for SMYD2 and SMYD3 crystal structures. (PDF)

S3 Table *In Vitro* and *In Vivo* DMPK Results. (PDF)

S4 Table SMYD3 Inhibition constants for EPZ028862. (PDF)

S5 Table Proliferation IC_50_s and KRAS mutant status of assorted lung cancer cell lines. (PDF)

S6 Table Cell panel screening with SMYD2 or SMYD3 CRISPR-Cas9 knockout and SMYD2 and SMYD3 inhibitor treatment. Proliferation IC_50_s for EPZ039527, LLY-507, EPZ028862 (2D), EPZ028862 (3D) for a panel of 240 cancer cell lines. LogP values from CRISPR pooled screening for SMYD2 and SMYD3 sgRNAs for a panel of 313 cell lines.
(XLSX)
