# Supplementary material for: Small molecule inhibitors and CRISPR/Cas9 mutagenesis demonstrate that SMYD2 and SMYD3 activity are dispensable for autonomous cancer cell proliferation
Source: PLoS One. 2018 Jun 1;13(6):e0197372. doi: 10.1371/journal.pone.0197372 (PMC5983452; doi:10.1371/journal.pone.0197372)
Supplement: S1 File — (PDF) [file pone.0197372.s001.pdf]

Supplemental Materials and Methods:

Compound synthesis

Many of the intermediates have previously been described in WO 2016040505, WO 2016040504, WO 2016040498 and WO 2016040508.

Unless otherwise noted, intermediates were characterized by LC-MS to confirm the mass matched the structure and carried on to the next step without further purification.

EPZ032597: N-(1-((1-benzyl-1H-pyrazol-4-yl)methyl)azetidin-3-yl)-1-cyclopropyl-1H-1,2,3-triazole-4-carboxamide

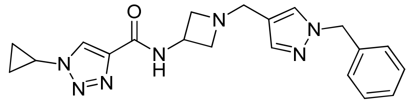

A mixture of N-(azetidin-3-yl)-1-cyclopropyl-1H-1,2,3-triazole-4-carboxamide (80 mg, 386 μmol), 1-benzyl-1H-pyrazole-4-carbaldehyde (86.2 mg, 463 μmol) and acetic acid (2.31 mg, 38.6 μmol) in methanol (10 mL) was stirred at room temperature for 30 min, then sodium cyanoborohydride (72.2 mg, 1.15 mmol) was added and stirred at 50 °C overnight. After completed, the solvent was removed in vacuo. The residue was diluted with Na_2CO_3 aqueous solution (10 mL) and extracted with ethyl acetate (15 mL x3). The organic layers were dried by anhydrous Na_2SO_4, filtered and concentrated. The crude product was purified by prep-TLC ((DCM: NH_3 in MeOH (7N)=20: 1) to give N-(1-((1-benzyl-1H-pyrazol-4-yl)methyl)azetidin-3-yl)-1-cyclopropyl-1H-1,2,3-triazole-4-carboxamide (110 mg, Yield: 75.8 %) as white solid.

ESI-LCMS (m/z): 400.3 $[M+Na]^+$;^{1}H NMR (400 MHz, CD_3OD) δ ppm: : 8.39 (s, 1H), 7.66 (s, 1H), 7.50 (s, 1H) , 7.35-7.29 (m, 3H), 7.23-7.21 (m, 2H), 5.32 (s, 2H), 4.65-4.62 (m, 1H), 4.00-3.97 (m, 1H), 3.71-3.67 (m, 2H), 3.61 (s, 2H), 3.28-3.19 (m, 2H), 1.27-1.21 (m, 4H) .
EPZ033294

EPZ033294: N-(1-((1-(4-chlorobenzyl)-1H-pyrazol-4-yl)methyl)azetidin-3-yl)-1-cyclopropyl-1H-1,2,3-triazole-4-carboxamide

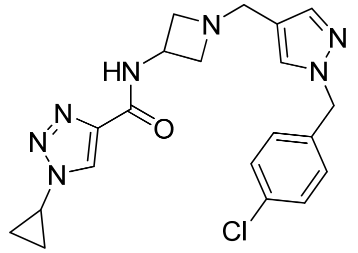

A mixture of N-(azetidin-3-yl)-1-cyclopropyl-1H-1,2,3-triazole-4-carboxamide (62 mg, 299 μmol), 1-(4-chlorobenzyl)-1H-pyrazole-4-carbaldehyde (intermediate D2, 65.9 mg, 299 μmol), sodium cyanoborohydride (56.3 mg, 897 μmol) in methanol (10 mL) was stirred at 60 °C for 16h. The mixture was cooled and concentrated. The residue was diluted with ethyl acetate, washed with water (10 mL) and brine (10 mLx2), dried over Na_2SO_4 and concentrated. The residue was purified by pre-HPLC to give N-(1-((1-(4- chlorobenzyl)-1H–pyrazol-4-yl)methyl)azetidin-3-yl)-1-cyclopropyl-1H-1,2,3- triazole-4-carboxamide (70 mg, 56.9 %) as as white solid.
ESI-LCMS (m/z): 412$[M+H]^+$;^{1}HNMR (400 MHz, CD_3OD) δ ppm: 8.38 (s, 1 H), 7.67 (s, 1 H), 7.50 (s, 1 H), 7.35 (d, J= 8.0 Hz, 2H), 7.21(d, J= 8.0 Hz , 2H), 5.31(s, 2H), 4.65-4.61(m, 1H), 4.00-3.96 (m, 1H), 3.71-3.67 (m, 2H), 3.61 (s, 2H), 3.22(t, J= 8.0 Hz, 1H), 1.30-1.24(m, 2H), 1.21-1.19(m, 2H).

Preparation of N-(azetidin-3-yl)-1-cyclopropyl-1H-1,2,3-triazole-4-carboxamide.

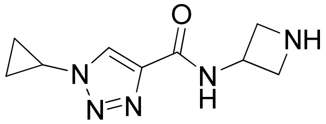

Step 1: Synthesis of ethyl 2-diazo-3-oxopropanoate

Oxalyl chloride (87.9 g, 693 mmol) was added to a cold solution of N,N-dimethylformamide (42.3 g, 578 mmol) in chloroform (150 mL) and the reaction was stirred at room temperature for 30 min, followed by heating at 40 °C for a further 1h. After chilling the reaction to -10 °C, ethyl 2-diazoacetate (63.0 g, 552 mmol) was added and the mixture was stirred at room temperature for 2h. The mixture was then concentrated and the residue was diluted with ether (200 mL), the solid was collected by filtration and dissolved in 10% aq. HOAc (200 mL), then stirred for a further 1h. The resulting mixture was extracted with **ethyl acetate** (300 mL x3) and the organic was washed with saturated Na_2CO_3 aq.(300 mL) and brine (300 mL), dried over Na_2SO_4, filtered and concentrated to give crude ethyl 2-diazo-3-oxopropanoate (27 g, 32.8 %) as red oil , which was used for next step without further purification.

^{1}H-NMR (400 MHz, CD_3OD) δ ppm: 9.67 (s, 1H), 4.33 (q, J = 7.2 Hz, 2H), 1.32 (t, J =7.2, 3H).

Step 2: Synthesis of ethyl 1-cyclopropyl-1H-1, 2, 3-triazole-4-carboxylate

A solution of ethyl 2-diazo-3-oxopropanoate (27 g, 189 mmol) in EtOH (100 mL) was added acetic acid (28.3 g, 472 mmol), then cyclopropanamine (10.7 g, 189 mmol) was added slowly, the mixture was stirred at room temperature overnight. Solvent was removed, the residue was added saturated Na_2CO_3 aq. until PH=8, the mixture was extracted with **ethyl acetate** (200 mL x3), washed with brine (100 mL), dried over Na_2SO_4, filtered and concentrated, the residue was purified by flash chromatography (PE:EA=2:1) to give crude ethyl 1-cyclopropyl-1H-1,2,3-triazole-4-carboxylate (18.5 g, 54.0 %) as yellow oil. ESI-LCMS (m/z): 182.2 $[M+H]^+$.

Step 3: Synthesis of 1-cyclopropyl-1H-1, 2, 3-triazole-4-carboxylic acid

A solution of ethyl 1-cyclopropyl-1H-1,2,3-triazole-4-carboxylate (18.5 g, 102 mmol) in THF (80 mL)/H_2O (40 mL) was added lithium hydroxide hydrate (4.5 g, 107 mmol) , the resulting mixture was stirred at room temperature for 3hr. Solvent was removed, the residue was dissolved in water (50 mL), extracted with ethyl acetate (100 mL) and discarded the organic, the water phase was added 2N HCl until pH=5, Then, the solution was extracted by DCM : MeOH = 10:1 (1.5 L). The dichloromethane layer was dried and concentrated to afford 6.3 g of 1-cyclopropyl-1H-1, 2, 3-triazole-4-carboxylic acid as white solid. The aqueous layer was concentrated to afford another 11.4 g crude product, which was used for next step without further purification. ESI-LCMS (m/z): 154.1$[M+H]^+$.

Step 4: Synthesis of tert-butyl 3-(1-cyclopropyl-1H-1, 2, 3-triazole-4- carboxam ido)azetidine-1-carboxylate

A solution of 1-cyclopropyl-1H-1,2,3-triazole-4-carboxylic acid (2 g, 13.0 mmol) in thionyl chloride (10 mL) was stirred at 65 °C for 2h. The reaction mixture was concentrated under reduced pressure. Then the reaction residue was diluted with DMF (5 mL) and added dropwise to the solution of tert-butyl 3-aminoazetidine-1- carboxylate (2.23 g, 13.0 mmol) and DIPEA (4.19 g, 32.5 mmol) in dichloromethane (15 mL) under 0 °C. The resulting mixture was stirred at room temperature overnight. Solvent was removed and the residue was diluted with **ethyl acetate** (200 mL), washed with water (10 mL x3) and brine (50 mL), dried over Na_2SO_4, filtered and concentrated, the residue was purified by flash chromatography (DCM: NH_3 in MeOH(7N)=100:0~50:1) to give tert-butyl 3-(1-cyclopropyl-1H-1,2,3-triazole-4- carboxamido)azetidine-1- carboxylate (3 g, 75.1 %) as a yellow solid. ESI-LCMS (m/z): 252.2 $[M-55]^+$.

Step 5: Preparation of N-(azetidin-3-yl)-1-cyclopropyl-1H-1, 2, 3-triazole-4- carboxamide

A solution of tert-butyl 3-(1-cyclopropyl-1H-1, 2 , 3-triazole-4-carboxamido)azetidine -1-carboxylate (3.0 g, 9.8 mmol) in HCl/MeOH (20 mL) was stirred at 50 °C for 2h. After completed, the solvent was removed in vacuo. The residue was dissolved in NH_3/MeOH (7 mol/L, 20 mL) and stirred for 30 min. Solvent was removed, the residue was purified by flash column chromatography (DCM: NH_3 in MeOH(7N)=100:0 ~30:1~15:1) to give N-(azetidin-3-yl)-1-cyclopropyl-1H-1,2,3-triazole-4-carboxamide (1.6 g, 80 %) as a white solid. ESI-LCMS (m/z): 208.1 $[M+H]^+$. ^{1}H-NMR (400 MHz, CD_3OD) δ ppm: 8.38 (s, 1H), 4.92-4.86 (m, 1H), 3.98-3.97 (m, 1H), 3.82-3.72 (m, 4H), 1.26-1.20 (m, 4H).

Preparation of 1-(4-chlorobenzyl)-1H-pyrazole-4-carbaldehyde

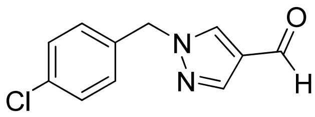

To a solution of 1H-pyrazole-4-carbaldehyde (70 mg, 728 µmol) in acetonitrile (5 mL) was added 1-(bromomethyl)-4-chlorobenzene (149 mg, 728 µmol) and cesium carbonate (472 mg, 1.45 mmol). The mixture was stirred at room temperature for 2h. The mixture was concentrated, diluted with ethyl acetate and water. The organic phase was washed with brine (10 mlx2), dried over Na_2SO_4 and concentrated to give 1-(4-chlorobenzyl)-1H- pyrazole-4-carbaldehyde (162 mg, 101 %) as a white solid, which was used in the next step without further purification. ESI-LCMS (m/z): 221$[M+H]^+$.

Preparation of Intermediate 1: 5-cyclopropylisoxazole-3-carboxylic acid

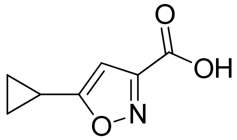

Step 1: Synthesis of ethyl 4-cyclopropyl-2,4-dioxobutanoate

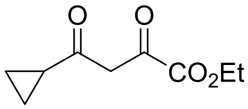

Into a 10-L 3-necked round-bottom flask purged and maintained with an inert atmosphere of nitrogen Na (164 g, 1.20 equiv) was added in portions to ethanol (5 L). A solution of $(CO_2Et)_2$ (869 g, 1.00 equiv) and 1-cyclopropylethan-1-one (500 g, 5.94 mol, 1.00 equiv) was added dropwise with stirring at 0-20°C. The resulting solution was stirred for 1 h at 20-30° C and then for an additional 1 h at 80°C. The resulting solution was diluted with 15 L of H_2O. The pH was adjusted to 2 with hydrochloric acid (12N). The resulting solution was extracted with ethyl acetate and the organic layers combined and washed with $NaHCO_3$ (sat. aq.). The extract was concentrated under vacuum yielding 820 g (crude) of ethyl 4-cyclopropyl-2,4-dioxobutanoate as yellow oil. TLC (ethyl acetate/petroleum ether =1/5): Rf = 0.5.

Step 2: Synthesis of ethyl 5-cyclopropylisoxazole-3-carboxylate

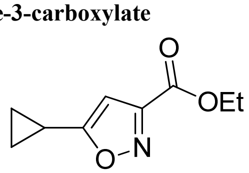

Into a 10 L round-bottom flask, was placed a solution of ethyl 4-cyclopropyl-2,4-dioxobutanoate (177 g) in ethanol (1.1 L) and NH_2OH-HCl (200 g). The resulting solution was stirred for 1 h at 20-30°C. The resulting solution was allowed to react, with stirring, for an additional 1 h at 80°C. The resulting mixture was concentrated under vacuum. The residue was purified on a silica gel column with ethyl acetate/petroleum ether (1/10). This resulted in 143 g (the two step yield was 66.3%) of ethyl 5-cyclopropylisoxazole-3-carboxylate as a yellow oil. TLC (ethyl acetate/petroleum ether =1/5): Rf = 0.2.

Step 3: Synthesis of 5-cyclopropylisoxazole-3-carboxylic acid

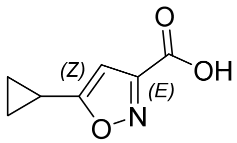

Into a 10-L round-bottom flask was placed ethyl 5-cyclopropylisoxazole-3-carboxylate (280 g, 1.55 mol, 1.00 equiv) and a solution of sodium hydroxide (74.3 g, 1.20 equiv) in water (4 L). The resulting solution was stirred for 1 h at room temperature. The resulting mixture was washed with ether. The pH value of the aqueous solution was adjusted to 2-3 with hydrochloric acid (12N). The resulting solution was extracted with ethyl acetate and the organic layers combined and concentrated under vacuum. This resulted in 220 g (93%) of 5-cyclopropylisoxazole-3-carboxylic acid as an off-white solid. LCMS (method A, ESI): RT = 1.99 min, m/z = 153.9 $[M+H]^+$. ^{1}H-NMR □ (300 MHz $CDCl_3$): 8.42(brs, 1H), 6.37(s, 1H), 2.16-2.05(m, 1H), 1.29-1.12(m, 2H), 1.12-0.99(m, 2H).

Preparation of EPZ-028862 N-((1R,3R,5S)-8-((1r,4R)-4-aminocyclohexylsulfonyl)-8-aza-bicyclo[3.2.1]octan-3-yl)-5-cyclopropylisoxazole-3-carboxamide hydrochloride

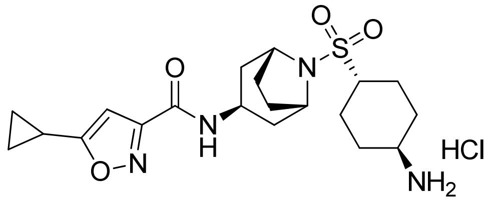

Step 1: Tert-butyl 3-amino-8-azabicyclo[3.2.1]octane-8-carboxylate

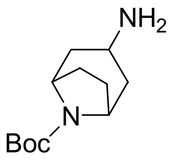

Into a 2000-mL 3-necked round-bottom flask was placed $HCOONH_4$ (42 g, 666.03 mmol, 30.00 equiv), acetic acid (1.3 g, 21.65 mmol, 1.00 equiv) and methanol (1.5 L). Then $NaBH_3CN$ (2.8 g, 44.56mmol, 2.00 equiv) was added into batch wise. This was followed by the addition of a solution of tert-butyl 3-oxo-8-azabicyclo[3.2.1]octane-8-carboxylate (5 g, 22.19mmol, 1.00 equiv) in methanol (100mL) dropwise with stirring at 25°C. The resulting solution was stirred for 12 h at 25°C. The resulting mixture was concentrated under vacuum. The resulting solution was diluted with 200mL of H_2O. The resulting solution was extracted with 3x200mL of ethyl acetate and the organic layers combined. The resulting mixture was washed with 1x200mL of brine (sat.). The mixture was dried over anhydrous sodium sulfate. The residue was applied onto a silica gel column with dichloromethane/methanol (9:1). This resulted in 4.8 g (90%) of tert-butyl 3-amino-8-azabicyclo[3.2.1]octane-8-carboxylate as colorless oil. LCMS (method D, ESI): RT=0.97 min, m/z =227.0 $[M+H]^+$.

Step 3: Tert-butyl (1R,3r,5S)-3-(5-cyclopropyl-1,2-oxazole-3-amido)-8-azabicyclo[3.2.1]octane-8-carboxylate and tert-butyl (1R,3s,5S)-3-(5-cyclopropyl-1,2-oxazole-3-amido)-8-azabicyclo[3.2.1]octane-8-carboxylate

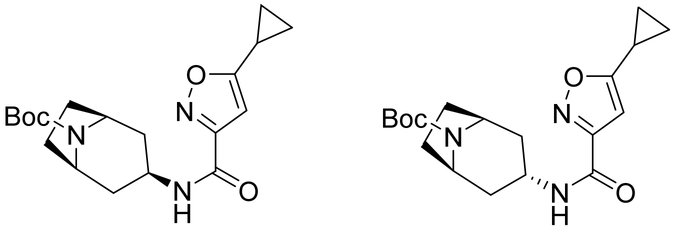

Into a 250-mL round-bottom flask was placed tert-butyl 3-amino-8-azabicyclo[3.2.1]octane-8-carboxylate (4 g, 17.67mmol, 1.00 equiv), 5-cyclopropyl-1,2-oxazole-3-carboxylic acid (2.7 g, 17.63mmol, 1.00 equiv), HATU (10 g, 26.30mmol, 1.50 equiv), DIEA (5.7 g, 44.10mmol, 2.50 equiv), and DMF(100mL). The resulting solution was stirred for 12 h at 25°C. The reaction was then quenched by the addition of 100mL of water. The resulting solution was extracted with 3x100mL of ethyl acetate and the organic layers combined. The resulting mixture was washed with 1x100mL of brine. The mixture was dried over anhydrous sodium sulfate and concentrated under vacuum. The residue was purified on a silica gel column with ethyl acetate/petroleum ether (1:4). The

product (4.0g) was further purified by Prep-SFC with the following conditions (prep SFC 350): Column, Phenomenex Lux 5u Cellulose-3, 5*25cm, 5um; mobile phase, CO_2(80%), methanol(20%); Detector, UV220nm. This resulted in 800 mg (13%) of tert-butyl (1R,3s,5S)-3-(5-cyclopropyl-1,2-oxazole-3-amido)-8-azabicyclo[3.2.1]octane-8-carboxylate as a yellow solid. ^{1}H NMR (400 MHz, $CDCl_3$) □: 6.53 (d, *J=8.0Hz*, 1H), 6.33(s, 1H), 4.41-4.58(m, 1H), 4.24-4.32(m, 2H), 1.95-2.11 (m, 5H), 1.80-1.84(m, 2H), 1.57-1.63(m, 2H), 1.50(s, 9H), 1.16-1.28(m, 2H), 0.95-1.06(m, 2H). LCMS (method D, ESI): RT=2.33 min, *m/z* =362.0 $[M+H]^+$ and 1.4 g (22%) of tert-butyl (1R,3r,5S)-3-(5-cyclopropyl-1,2-oxazole-3-amido)-8-azabicyclo[3.2.1]octane-8-carboxylate as a yellow solid. ^{1}H NMR (400 MHz, $CDCl_3$) □: 7.21-7.23(d, *J=7.6Hz*, 1H), 6.34(s, 1H), 4.27-4.33(m, 3H), 2.25-2.31(m, 2H), 2.07-2.14 (m, 3H), 1.91-1.95(m, 2H), 1.76-1.80(m, 2H), 1.49(s, 9H), 1.16-1.28(m, 2H), 0.95-1.06(m, 2H). LCMS (method D, ESI): RT=2.43 min, *m/z* =362.0 $[M+H]^+$.

Step 4: N-((1R,3r,5S)-8-aza-bicyclo[3.2.1]octan-3-yl)-5-cyclopropylisoxazole-3-carboxamide hydrochloride

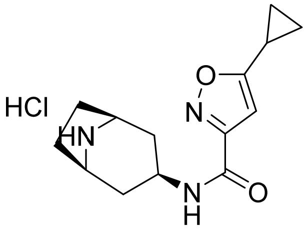

Into a 50-mL round-bottom flasks was placed tert-butyl (1R,3r,5S)-3-(5-cyclopropyl-1,2-oxazole-3-amido)-8-azabicyclo[3.2.1]octane-8-carboxylate (600 mg, 1.66mmol, 1.00 equiv). This was followed by the addition of 10 mL of 1,4-dioxane into each flask. Then hydrogen chloride was introduced into the two mixtures. The resulting solutions were stirred for 2 h at 25°C. The resulting mixtures were concentrated under vacuum. This resulted in 480 mg (97%) of N-((1R,3r,5S)-8-aza-bicyclo[3.2.1]octan-3-yl)-5-cyclopropylisoxazole-3-carboxamide hydrochloride LCMS (method D, ESI): RT=0.97 min, m/z =262.0 $[M+H]^+$.

Step 5: 5-cyclopropyl-N-((1R,3r,5S)-8-(4-oxocyclohexylsulfonyl)-8-aza-bicyclo[3.2.1]octan-3-yl)isoxazole-3-carboxamide

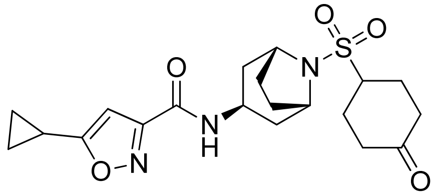

Into a 2-L 3-necked round-bottom flask was placed a solution of N-((1R,3r,5S)-8-azabicyclo[3.2.1]octan-3-yl)-5-cyclopropylisoxazole-3-carboxamide hydrochloride (20 g, 67.16 mmol, 1.00 equiv) in dichloromethane (800 mL). Then DIEA (43 g, 332.71 mmol, 5.00 equiv) was added, followed by the addition of 4-oxocyclohexane-1-sulfonyl chloride (14.45 g, 73.48 mmol, 1.10 equiv) in portions over 5.5 hr (0.1 equiv for each portion). The resulting solution was stirred overnight at 20°C. The reaction mixture was washed with dilute hydrochloric acid (1N, 200 mL). Then the organic phase was washed with $NaHCO_3$ (sat. 200 mL) and brine (sat. 200 mL) respectively. The organic phase was dried over anhydrous Na_2SO_4 and concentrated under vacuum. This resulted in 19 g (64%) of 5-cyclopropyl-*N*-((1R,3r,5S)-8-(4-oxocyclohexylsulfonyl)-8-aza-bicyclo[3.2.1]octan-3-yl)isoxazole-3-carboxamide as a yellow solid. ^{1}H-NMR (300 MHz, $CDCl_3$): δ 7.14(d, *J* = 9 Hz, 1H), 6.34(s, 1H), 4.37-4.25(m, 3H), 3.36-3.27(m, 1H), 2.65-2.15(m, 10H), 2.13-1.9(m, 7H), 1.20-1.10(m, 2H), 1.05-0.95(m, 2H) *ppm*. LCMS (method C, ESI): RT = 0.88 min, *m/z* = 422.2 $[M+H]^+$.

Step 6: N-((1R,3r,5S)-8-(((1r,4R)-4-aminocyclohexyl)sulfonyl)-8-azabicyclo[3.2.1]octan-3-yl)-5-cyclopropylisoxazole-3-carboxamide hydrochloride

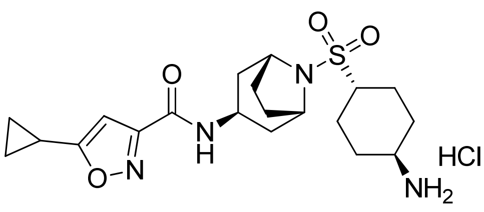

Into a 5-L round-bottom flask was placed a solution of 5-cyclopropyl-*N*-((1R,3r,5S)-8-(4-oxocyclohexylsulfonyl)-8-aza-bicyclo[3.2.1]octan-3-yl)isoxazole-3-carboxamide (3 g, 7.12 mmol, 1.00 equiv) in methanol (3 L), then $HCOONH_4$ (17.6 g, 279.12

mmol, 40.00 equiv) and acetic acid (852 mg, 14.19 mmol, 2.00 equiv) were added. After stirred for 30 min at 25°C, $NaBH_3CN$ (895 mg, 14.24 mmol, 2.00 equiv) was added portion-wise. The resulting solution was stirred for 30 min at 25°C. The reaction mixture was concentrated under vacuum. The resulting solid was extracted with ethyl acetate (100 mLx5). The combined organic extracts were concentrated and the residue purified by flash chromatography (DCE: MeOH = 10:1). The crude product was further purified by Prep-HPLC with the following conditions: Column, X Bridge C18, 19*150 mm, 5 um; mobile phase, Mobile Phase A: Water/0.05% TFA, Mobile Phase B: ACN; Flow rate: 20 mL/min; Detector, 254 nm. The fractions containing product were combined and concentrated. They were then treated with hydrochloric acid (12N, 1 mL) and concentrated again under vacuum. This resulted in 1.0 g (31%) of N-((1R,3R,5S)-8-((*1r,4R*)-4-aminocyclohexylsulfonyl)-8-aza-bicyclo[3.2.1]octan-3-yl)-5-cyclopropylisoxazole-3-carboxamide hydrochloride as a light yellow solid. ^{1}H-NMR (300 MHz, D_2O): δ 6.29(s, 1H), 4.21-4.00(m, 3H), 3.28-3.10(m, 2H), 2.30-2.05(m, 7H), 2.05-1.87(m, 6H), 1.65-1.35(m, 4H), 1.12-1.00(m, 2H), 0.95-0.84(m, 2H). LCMS (method D, ESI): RT = 0.89 min, *m/z* =423.1 $[M+H]^+$.

ADME methods

Results are summarized in Table S3.

Caco-2 permeability. Caco-2 cells (obtained from the ATCC), between passage numbers 40 to 60, were seeded onto Millipore Multiscreen plates at $1x10^5$ cells/cm^2 in Dulbecco's Modified Eagle Medium. Media was changed every two or three days. On Day 20, the permeability study was performed using Hanks Balanced Salt Solution (HBSS) pH 7.4 buffer with 25 mM HEPES and 4.45 mM glucose at 37°C as the medium. Incubations were carried out in an atmosphere of 5% CO_2 with a relative humidity of 95% at 37°C. The monolayers were prepared by rinsing both basolateral and apical surfaces twice with HBSS at 37°C. Cells were then incubated with HBSS in both apical and basolateral compartments for 40 min to stabilize physiological parameters. HBSS was then removed from the apical or basolateral compartment and replaced with 10 µM test compound dosing solutions (1% final DMSO). The pH of supplemented HBSS was 6.5 and 7.5 in the apical and basolateral compartments, respectively. The apical compartment inserts were then placed into 'companion' plates containing fresh HBSS. At 120 min the companion plate was removed and apical and basolateral samples diluted for analysis by LC-MS/MS. Permeability was assessed in duplicate, on two different occasions. On each plate atenolol and propanolol were run as controls. Compounds were quantified by LC-MS/MS analysis using a 5 point calibration curve. The starting concentration (C_0) was determined from the dosing solution and experimental recovery calculated from C_0 and both apical and basolateral compartment concentrations. The permeability coefficient (P_{app}) for each compound was calculated as: $(dQ/dt)/(C_0 xA)$, where dQ/dt is the rate of permeation of the drug across the cells, C_0 is the donor compartment concentration at time zero and A is the area of the cell monolayer.

Plasma protein binding. Plasma protein binding was assessed by equilibrium dialysis utilizing the HT-dialysis cell format with a cellulose semi-permeable membrane (molecular weight cut-off of 5000 Da). Solutions of test compounds (1 µM, 0.5% final DMSO concentration) were prepared in isotonic phosphate buffer (pH 7.4) and 100% pooled male CD-1 mouse plasma. The buffer solution was added to one side of the membrane and the plasma solution to the other side. Incubations were performed in duplicate for 16 h, at 37°C, in order to allow the compounds to reach equilibrium. Haloperidol was incubated in parallel as the control compound for each species. At the end of the incubation time, samples were taken from both sides of the membrane. Following protein precipitation, the samples were analyzed by LC-MS/MS using two sets of calibration standards for protein free (7 points) and protein containing solutions (6 points). Samples were quantified using standard curves prepared in the equivalent matrix. The fraction unbound in plasma (f_u) was calculated using the following equation:

$$f_u^{corrected} = \frac{PF}{\{(PC - PF) \times V_{Correction}\} + PF}$$

Where, PC = sample concentration in protein containing side, PF = sample concentration in protein free side, and $V_{Correction}$ = correction factor for the volume shift i.e. ratio of the volume of the protein after dialysis to that before dialysis.

Liver microsome (LM) stability. Pooled LM from CD-1 mice, SD rats, beagle dogs, cyno-monkeys, and humans were used (male only for animal LM and mixed gender for human LM). LM of each species (0.5 mg/mL), 0.1 M phosphate buffer pH 7.4 and 1 µM test compound (0.25% DMSO final) were pre-incubated in duplicate at 37°C prior to the addition of 1 mM NADPH to initiate the reaction. The final incubation volume was 50 µL. Negative control incubations were included where phosphate buffer was added instead of NADPH. Positive controls were diazepam and diphenhydramine for mice and rats, dextromethorphan and verapamil for dogs and humans, and quinidine and verapamil for monkeys. Each compound was incubated for 0, 5, 15, 30 and 45 min. The negative controls were incubated for 45 min only. The reactions were stopped by transferring 25 µL of incubate to 50 µL methanol at the appropriate time points. Termination plates were centrifuged at 2,500 rpm for 20 min at 4°C to precipitate the protein and metoprolol (internal standard) was added to the sample supernatants prior to LC-MS/MS analysis. *In vitro* $t_{½}$ values were determined by plotting the natural logarithm of the analyte/internal standard peak area ratios as a function of time, with the slope of the linear regression (-k) converted to *in vitro* $t_{½}$ value where $t_{½} = -0.693/k$. Subsequently, intrinsic CL (CL_{int}) was

calculated as: (incubation volume/microsomal protein)x0.693/$t_{½}$ and scaled CL values were obtained using the well-stirred venous equilibration model.

Hepatocyte stability. Cyropreserved hepatocytes from CD-1 mice, SD rats, beagle dogs, cyno-monkeys, and humans (male only for animal hepatocytes and mixed gender for human hepatocytes) were used. Williams E media supplemented with 2 mM L-glutamine and 25 mM HEPES and test compound (final substrate concentration 1 µM; final DMSO concentration 0.25 %) were pre-incubated at 37 °C prior to the addition of a suspension of cryopreserved hepatocytes (final cell density 0.5 x 10^6 viable cells/mL in Williams E media supplemented with 2 mM L-glutamine and 25 mM HEPES) to initiate the reaction. The final incubation volume was 500 µL. A control incubation was included for each compound tested where lysed cells were added instead of viable cells. Two control compounds were included with each species. The reactions were stopped by transferring 50 µL of incubate to 100 µL methanol containing internal standard at the appropriate time points (0, 5, 10, 20, 40 and 60 min). The control (lysed cells) was incubated for 60 min only. The termination plates were centrifuged at 2500 rpm at 4 °C for 30 min to precipitate the protein and metoprolol (internal standard) was added to the sample supernatants prior to LC-MS/MS analysis. *In vitro* $t_{½}$ values were determined by plotting the natural logarithm of the analyte/internal standard peak area ratios as a function of time, with the slope of the linear regression (-k) converted to *in vitro* $t_{½}$ value where $t_{½}$ = -0.693/k. Subsequently, intrinsic CL (CL_{int}) was calculated as: (incubation volume/number of hepatocytes)x0.693/$t_{½}$ and scaled CL values were obtained using the well-stirred venous equilibration model..

Inhibition of Cytochrome P450 (CYP) isoforms: Two types of inhibition were evaluated: 1) co-incubation of test compound with respective CYP subsrate (CYPs 1A2, 2C19, 2C9, 2D6 and 3A4) to determined the IC_{50}; 2) preincubation with test compound for 30 min prior to addtion of CYP3A4 substrate midazolam) to determine IC_{50} shift.

1. *Inhibition of specific CYP isoforms in a non-preincubation manner (IC_{50} Determination):* Six concentrations of EZP028862 (0.1, 0.25, 1, 2.5, 10, 25 µM; final DMSO concentration 0.25 %) was incubated with human LM and NADPH in the presence of a CYP isoform-specific probe substrate. The respecitve metabolites of specific substrates were monitored by LC-MS/MS. A decrease in the formation of the metabolite compared to the vehicle control was used to calculate an IC_{50} value (test compound concentration which produces 50 % inhibition). Experimental conditions for evaluating inhibition of individual CYP isoforms are provided in the table below.

CYP Isoform	Substrate (Concentration)	Metabolite	HLM concentration;Incubation time
CYP1A2	phenacetin (30 µM)	acetaminophen	0.25 mg/mL; 5 min
CYP2C19	mephenytoin (25 µM)	4'-OH-(S) mephenytoin	0.5 mg/mL; 60 min
CYP2C9	tolbutamide (120 µM)	4-OH tolbutamide	1 mg/mL; 60 min
CYP2D6	dextromethorphan (5 µM)	dextrophan	0.5 mg/mL; 5 min
CYP3A4	midazolam (2.5 µM)	1'-OH midazolam	0.1 mg/mL; 5 min
CYP3A4	testosterone (50 µM)	6-OH testosterone	0.5 mg/mL; 5 min

2. *Time-dependent inhibition of CYP3A4(Midazolam; IC_{50} Shift):* Six concentrations of EZP028862 (0.1, 0.25, 1, 2.5, 10, 25 µM; final DMSO concentration 0.25 %, final microsome concentration 0.1 mg/mL) were either pre-incubated for 30 min in the absence and presence of NADPH or undergo a 0 min pre-incubation. The probe substrate midazolam (2.5 µM) and NADPH (1 mM) were then added (final DMSO concentration 0.26 %) and the samples incubated for 5 min at 37 °C. The selective time dependent CYP3A4 inhibitor, mifepristone, was screened alongside the test compounds as a positive control. The reactions were terminated by the addition of an aliquot of the incubation into methanol. The samples were centrifuged at 2500 rpm for 30 min at 4 °C, and aliquots of the supernatant were diluted with formic acid in deionised water (final concentration 0.1 %) containing internal standard. 1-hydroxymidazolam was monitored by LC-MS/MS and a decrease in the formation of the metabolite compared to the vehicle control was used to calculate an IC_{50} value for each of the experimental conditions. The 0 min pre-incubation acted as a control for measuring the reversible inhibition and was used to compare against the 30 min pre-incubation minus NADPH. A ratio of the calculated IC_{50} for 30 min pre-incubation minus NADPH and 30 min pre-incubation plus NADPH was used to calculate the fold shift in IC_{50}**.**

Mouse, rat, dog and monkey pharmacokinetic studies. The studies were performed in accordance with the AAALAC International guidelines standards.

1. Mouse PK: for EPZ028862, male CD-1 mice (27-31 g; Beijing HFK Bio-Technology Co., LTD. n=3/group) were administered intravenously (1 mg/kg in saline, tail vein injection, 2 mL/kg) and orally (5 mg/kg in 0.5% MC/0.1% Tween 80, oral gavage, 10 mL/kg). Animals were weighed prior to dose administration and had free access to water and food. At pre-specified time intervals up to 24 hours post-dose, blood samples (approximately 30 µL) were collected from saphenous vein into polypropylene micro centrifuge tubes (containing 2 µL of 1000 IU/ml Heparin-Na as anticoagulant) and stored on wet ice until processed to plasma by centrifugation (2000 rpm

for 5 min at 4°C) within 30 min of collection. In the same study, urine was collected for 24 hours. For EPZ033294, male CD-1 mice (25-34 g; Envigo, South Eston, MA, USA; n=3/group) were administered intravenously (2 mg/kg in ethanol:saline (10:90) solution, tail vein injection, 2 mL/kg) and orally (100 mg/kg in ethanol:saline (20:80) solution, oral gavage, 10 mL/kg). Animals were weighed prior to dose administration and fasted approximately 2 hours prior to dosing. Food was returned 2 hours after dosing. At pre-specified time intervals up to 24 hours post-dose, blood samples (approximately 35 to 40 uL) were collected into calibrated Microvette tubes (K_2EDTA as anticoagulant) via tail snip and stored on wet ice until processed to plasma by centrifugation (3500 rpm at 5°C) within 1 hour of collection.

2. Rat PK: male Sprague Dawley rats (190-254 g; Vital River Laboratories Co., Ltd. n=3/group) were administered EPZ028862 intravenously (1 mg/kg in 5%NMP/5% Solutol/90%Saline, tail vein injection, 1 mL/kg) and orally (5 mg/kg in 5%NMP/5% Solutol/90%Saline, oral gavage, 5 mL/kg). Animals were weighed prior to dose administration and fasted overnight. Food was returned 4 hours after dosing. At pre-specified time intervals up to 24 hours post-dose, blood samples (approximately 300 µL) were collected into tubes (containing K_2EDTA as anti-coagulant) via jugular vein catheter and stored on wet ice until processed to plasma by centrifugation (2000 rpm for 5 min at 4°C) within 30 min of collection. In the same study, urine was collected for 24 hours.
3. Dog PK: male beagel dogs (6.0-6.5 kg, Beijing Marshall Biotechnology Co., Ltd. cross-over n=3/group) were administered EPZ028862 intravenously (1 mg/kg in 5% ethanol aquous solution, saphenous vein injection, 1 mL/kg) and orally (5 mg/kg in 0.1% Tween in 0.5% methylcellulose water solution, oral gavage, 5 mL/kg). Animals were weighed prior to dose administration and fasted overnight. Food was returned 4 hours after dosing. At pre-specified time intervals up to 24 hours post-dose, blood samples (approximately 1 mL) were collected into tubes (containing K_2EDTA as anti-coagulant) via jugular vein catheter and stored on wet ice until processed to plasma by centrifugation (2000 rpm for 10 min at 2-8°C) within 30 min of collection. In the same study, urine was collected for 24 hours.
4. Monkey PK: male cynomolgous monkeys (3.6-4.1 kg, Guangxi Guidong, cross-over n=3/group) were administered EPZ028862 intravenously (1 mg/kg in 5% ethanol aquous solution, saphenous vein injection, 1 mL/kg) and orally (5 mg/kg in 0.1% Tween in 0.5% methylcellulose water solution, oral gavage, 5 mL/kg). Animals were weighed prior to dose administration and fasted overnight. Food was returned 4 hours after dosing. At pre-specified time intervals up to 24 hours post-dose, blood samples (approximately 1 mL) were collected into tubes (containing K_2EDTA as anti-coagulant) via jugular vein catheter and stored on wet ice until processed to plasma by centrifugation (2000 rpm for 10 min at 2-8°C) within 30 min of collection. In the same study, urine was collected for 24 hours.

The plasma and urine samples collected in all PK studies were stored at -70 ± 10°C prior to protein precipitation and LC-MS/MS analysis. Standard calibration curves were constructed by analyzing a series of control plasma aliquots or urine containing internal standard and test compound. Four levels of quality controls were also included in the analysis. The concentration of test compound in each unknown sample was determined by solving the linear calibration curve equation for each corresponding drug/internal standard ratio. Data were analyzed using noncompartmental methods (Phoenix WinNonlin 6.3.0).

Some specifics of LC-MS/MS methods applied to respective PK studies are provided in the table below.

PK Study	Test Compound	Internal Standard	Standard Curve Range; Matrix
Mouse PK	EPZ028862	Omeprazole	2–5000 ng/mL; plasma and urine
Mouse PK	EPZ033294	Carbutamide	0.5–5000 ng/mL; plasma
Rat PK	EPZ028862	Omeprazole	2–1000 ng/mL (plasma); 2–5000 ng/mL (urine)
Dog PK	EPZ028862	Omeprazole	2–1000 ng/mL (plasma); 2–5000 ng/mL (urine)
Monkey PK	EPZ028862	Omeprazole	2–1000 ng/mL (plasma); 2–5000 ng/mL (urine)

Supplemental Results:SMYD2 compound characterization

SMYD2 steady-state kinetics were measured to determine the apparent K_M values for H3, 1-29 peptide and SAM substrates. The dependence of the initial velocity on H3, 1-29 concentration was well described by eq 1 giving an apparent K_M value of 66 ± 11 nM (Figure S8A). SAM displays a high affinity for SMYD2 requiring a tight-binding treatment of the data using eq 2 giving an apparent K_M value of 0.34 ± 0.07 nM. As this K_M value is approximately half of the concentration of enzyme used in the assay, it is an upper limit for the true K_M value (Figure S8B). Higher apparent SAM K_M values of 30-60 nM have been reported though,

these studies used a p53 peptide substrate and were run at pH 9.0 [1, 2]. In support of a high affinity between SMYD2 and SAM, Ferguson has reported that SMYD2 co-purifies with SAM [3]. To determine the mechanism of SMYD2 inhibition with respect to peptide, H3,1-29 and EPZ033294 or EPZ032597 were simultaneously varied. IC_{50} values for both EPZ032597 and EPZ033294 were independent of the concentration of peptide substrate consistent with noncompetitive inhibition with K_i values of 21.5 ± 1.5 nM and 3.9 ± 0.4 nM respectively (Figures S9 and 2B). Since concentrations of SAM below its K_M value could not be tested for SMYD2, the corresponding dual titration of inhibitor and SAM was not performed. Instead, the SAM-dependence of inhibition was investigated by comparing IC_{50} values at 20 and 200 nM SAM using 1 nM SMYD2 and 60 nM H3,1-29. The IC_{50} values were 2.8 ± 0.4 nM and 3.2 ± 0.5 nM for EPZ033294 and 21 ± 2 nM and 25 ± 5 nM for EPZ032597 for 20 nM and 200 nM SAM respectively (data not shown). Such independence of inhibition over a 10-fold range of SAM concentrations is inconsistent with a competitive mechanism. In the absence of data at subsaturating concentrations of SAM, noncompetitive and uncompetitive mechanisms cannot be distinguished for these compounds. Importantly, these data indicate that EPZ032597 and EPZ033294 are effective inhibitors of SMYD2 at saturating concentrations of SAM which is likely to be the case *in vivo* based on the K_M value for SAM.The 2.69 Å resolution crystal structure of EPZ033294 solved in complex with SMYD2 and SAM unexpectedly revealed two molecules bound in the peptide binding site (Figure S1A). The cyclopropyl triazole moiety of one molecule of EPZ033294 binds in the lysine channel and is within 3.9 Å of the methyl group of SAM (Figure 2C). The triazole ring forms a π-stacking interaction with Phe184 while the amide linker forms a hydrogen bond with the backbone amide of Thr184. The azetidine ring nitrogen forms a hydrogen bond interaction with the side chain of Glu187. Interestingly, the compound induces a pocket into which the hydrophobic tail of the molecule binds (Figure S1B). The loop containing residues 187-192 shifts 1.5 Å and residues Met412 and Tyr 422 also shift to accommodate the aromatic tail. This binding mode is unique amongst published SMYD2 inhibitors (Figure S1C). Compared to the molecule engaged in the lysine channel, the second, EPZ033294, makes significantly fewer polar and van der waals interactions with the protein. The chlorobenzyl group of the compound binds in a hydrophobic region utilized by other SMYD2 inhibitors and the peptide substrate. The cyclopropyl triazole moiety engages in a π-stacking interaction with Arg 253 and the amide carbonyl makes a hydrogen bond with the backbone NH of Asp 242. It was hypothesized that the first molecule binding mode is representative of the active inhibitor binding mode while the second binding site is an artifact of the high concentration of compound (4 mM) used in the crystal soaking experiments necessary to produce the complex structure. ITC experiments were performed in order to get a stoichiometry value for the binding of EPZ033294 to SMYD2 (Figure S10A). The dissociation constant K_D for EPZ033294 binding to SMYD2 in the ITC assay was found to be 8.3 ± 8.2 nM where the error represents the standard deviation of the mean value as determined from three test occasions. This K_D is in agreement with the value of 5 nM determined by SPR (Figure S10B). In all ITC experiments the binding isotherms approached step functions due to tight binding of EPZ033294. Thus, curve fits for K_D were non-ideal as reflected by the large standard deviation of the mean K_D value. The stoichiometry of binding was found to be close to 1:1 (N = 0.7 ± 1) consistent with the hypothesis that the observed 2:1 stoichiometry seen in the crystal structure is most likely due to crystallization conditions. EPZ033294 was profiled across an *in vitro* ADME panel to further establish its potential utility as an *in vivo* tool compound (Table S3). EPZ033294 was shown to be both permeable and an efflux substrate in the Caco-2 assay. EPZ033294 was not highly bound in either mouse or human plasma protein binding where the fractions unbound were 0.26 and 0.13 respectively. The microsomal stability of EPZ033294 in both mouse and human hepatocytes was good justifying exploration in an *in vivo* rodent PK experiment. Dosing male CD-1 mice 2 mg/kg IV and 10 mg/kg PO established that EPZ033294 had excellent bioavailability and a long $t_{1/2}$ suggesting it would be an excellent choice for mouse *in vivo* exploratory studies to better understand the utility of SMYD2 inhibition.

SMYD3 compound characterization

The mechanism of EPZ028862 inhibition of SMYD3 was determined versus both substrates. The IC_{50} value for EPZ028862 was independent of MEKK2 concentration and best described by noncompetitive inhibition with a K_i value of 1.49 ± 0.15 nM (Figure S11A, Table S4). EPZ028862 inhibition of SMYD3 increased modestly with increasing SAM concentration and was best described by mixed-type inhibition with a K_i value of 2.64 ± 0.48 nM and an αK_i value of 0.65 ± 0.13 nM (Figure S11B, Table S4). The mechanism of inhibition by EPZ028862 is the same as the recently described oxindole inhibitors EPZ030456 and EPZ031686 [4]. As with EPZ030456 and EPZ031686, EPZ028862 displays noncompetitive inhibition versus MEKK2 despite observation of compound binding in the substrate lysine site. It is hypothesized that the binding affinity of MEKK2 for SMYD3 is largely derived from protein interactions outside of the lysine site such that these small molecule inhibitors cannot displace MEKK2.The binding kinetics of EPZ028862 to SMYD3 was further explored using SPR. EPZ028862 binding to the protein showed significant residence time, therefore, single-cycle kinetics methods were used to determine the kinetic values. A subnanomolar dissociation constant, K_D = 0.35 ± 0.10 nM was measured (Figure S12) and this result was consistent with the IC_{50}

= 1.3 nM as measured in the functional enzyme assay. The 1.42 Å resolution crystal structure of EPZ028862 was solved in a ternary complex with SMYD3 and SAM. The cyclopropyl isoxazole headgroup makes several hydrophobic and electrostatic interactions with the lysine pocket of SMYD3 (Figure 4B). The cyclopropyl moiety is 3.8 Å from the SAM methyl group. The aromatic isoxazole ring forms a □from the SAM methyl group. The aromatic isoxazole ring forms a y complex with SMYD3 and SAM. The cyclopropyl isoxazole headgroup makes several hydrophobic and electrostatic interactions with the lysine pocket of SMYD3 (Figure" timestamp="1473192727">15</n substrate [5], [6] . The sulfonamide and terminal amine groups make extensive water mediated interactions with the protein (Glu192, Lys297, Asp332, His366, Leu240) that are visible due to the high resolution of this crystal structure. Overall, the position of EPZ028862 in the SMYD3 active site is similar to that of EPZ030456 [4] despite the different moieties binding in the lysine channel and alterations to the tail. The amide linker, bridged ring system and sulfonamide superimpose well between the structures, and the basic amine of both compounds is positioned similarly (0.8 Å) within the binding site (Figure S13).EPZ028862 was profiled across an *in vitro* ADME panel to further credential its utility as an *in vivo* tool compound (Table S3). EPZ028862 was shown to be both permeable and an efflux substrate in the Caco-2 assay. EPZ028862 was profiled for its plasma binding potential across several species. EPZ028862 was generally not highly bound demonstrating a fraction unbound of 0.51, 0.64, 0.82 and 0.71 for mouse, rat, dog and human respectively, although EPZ028862 was highly bound in monkey plasma proteins. The microsomal and hepatocyte stability of EPZ028862 across mouse, rat, dog, monkey and human assays was excellent justifying exploration in an *in vivo* rodent PK experiment. Dosing male CD-1 mice 1 mg/kg IV and 5 mg/kg PO established that EPZ033294 showed 100% bioavability and a long $t_{1/2}$ suggesting it would be an excellent choice for mouse *in vivo* exploratory studies. EPZ028862 was also examined in rat, dog and non-human primate PK studies where the excellent mouse bioavailablity and overall PK profile was recapitulated. This establishes EPZ028862 as an excellent tool compound to explore the *in vivo* biology of SMYD3 across multiple species.

Supplemental References

1. Wu J, Cheung T, Grande C, Ferguson AD, Zhu X, Theriault K, et al. Biochemical characterization of human SET and MYND domain-containing protein 2 methyltransferase. Biochemistry. 2011;50(29):6488-97. Epub 2011/06/18. doi: 10.1021/bi200725p. PubMed PMID: 21678921.

2. Eggert E, Hillig RC, Koehr S, Stockigt D, Weiske J, Barak N, et al. Discovery and Characterization of a Highly Potent and Selective Aminopyrazoline-Based in Vivo Probe (BAY-598) for the Protein Lysine Methyltransferase SMYD2. J Med Chem. 2016;59(10):4578-600. Epub 2016/04/15. doi: 10.1021/acs.jmedchem.5b01890. PubMed PMID: 27075367; PubMed Central PMCID: PMC4917279.

3. Ferguson AD, Larsen NA, Howard T, Pollard H, Green I, Grande C, et al. Structural basis of substrate methylation and inhibition of SMYD2. Structure. 2011;19(9):1262-73. Epub 2011/07/26. doi: 10.1016/j.str.2011.06.011. PubMed PMID: 21782458.

4. Mitchell LH, Boriack-Sjodin PA, Smith S, Thomenius M, Rioux N, Munchhof M, et al. Novel Oxindole Sulfonamides and Sulfamides: EPZ031686, the First Orally Bioavailable Small Molecule SMYD3 Inhibitor. ACS Med Chem Lett. 2016;7(2):134-8. Epub 2016/03/18. doi: 10.1021/acsmedchemlett.5b00272. PubMed PMID: 26985287; PubMed Central PMCID: PMC4753551.

5. Van Aller GS, Graves AP, Elkins PA, Bonnette WG, McDevitt PJ, Zappacosta F, et al. Structure-Based Design of a Novel SMYD3 Inhibitor that Bridges the SAM-and MEKK2-Binding Pockets. Structure. 2016;24(5):774-81. Epub 2016/04/14. doi: 10.1016/j.str.2016.03.010S0969-2126(16)00088-5 [pii]. PubMed PMID: 27066749.

6. Fu W, Liu N, Qiao Q, Wang M, Min J, Zhu B, et al. Structural Basis for Substrate Preference of SMYD3, a SET Domain-containing Protein Lysine Methyltransferase. The Journal of biological chemistry. 2016;291(17):9173-80. Epub 2016/03/02. doi: 10.1074/jbc.M115.709832. PubMed PMID: 26929412; PubMed Central PMCID: PMC4861483.

7. Wang L, Li L, Zhang H, Luo X, Dai J, Zhou S, et al. Structure of human SMYD2 protein reveals the basis of p53 tumor suppressor methylation. The Journal of biological chemistry. 2011;286(44):38725-37. Epub 2011/09/02. doi: 10.1074/jbc.M111.262410. PubMed PMID: 21880715; PubMed Central PMCID: PMC3207477.

8. Jiang Y, Trescott L, Holcomb J, Zhang X, Brunzelle J, Sirinupong N, et al. Structural insights into estrogen receptor alpha methylation by histone methyltransferase SMYD2, a cellular event implicated in estrogen signaling regulation. J Mol Biol. 2014;426(20):3413-25. Epub 2014/03/07. doi: 10.1016/j.jmb.2014.02.019. PubMed PMID: 24594358.

9. Sweis RF, Wang Z, Algire M, Arrowsmith CH, Brown PJ, Chiang GG, et al. Discovery of A-893, A New Cell-Active Benzoxazinone Inhibitor of Lysine Methyltransferase SMYD2. ACS Med Chem Lett. 2015;6(6):695-700. Epub 2015/06/24. doi: 10.1021/acsmedchemlett.5b00124. PubMed PMID: 26101576; PubMed Central PMCID: PMC4468393.

10. Nguyen H, Allali-Hassani A, Antonysamy S, Chang S, Chen LH, Curtis C, et al. LLY-507, a Cell-active, Potent, and Selective Inhibitor of Protein-lysine Methyltransferase SMYD2. J Biol Chem. 2015;290(22):13641-53. Epub 2015/04/01. doi: 10.1074/jbc.M114.626861

M114.626861 [pii]. PubMed PMID: 25825497; PubMed Central PMCID: PMC4447944.
