# Supplementary material for: Small molecule inhibitors and CRISPR/Cas9 mutagenesis demonstrate that SMYD2 and SMYD3 activity are dispensable for autonomous cancer cell proliferation
Source: PLoS One. 2018 Jun 1;13(6):e0197372. doi: 10.1371/journal.pone.0197372 (PMC5983452; doi:10.1371/journal.pone.0197372)

Figure S1: Crystal structure of EPZ033294 bound to SMYD2. (A) Two molecules of EPZ033294 (molecule 1 = cyan; molecule 2 = purple) were seen in the peptide binding site of SMYD2 (green). SAM (yellow) is show in stick representation. (B) The tail of EPZ033294 molecule 1(green) induces a hydrophobic pocket when compared to structures of SMYD2 (grey; PDB 3TG4 [7]); SMYD2-ER⍰ peptide (yellow; PDB 4O6F [8]) and SMYD2-p53 peptide (blue; PDB 3S7F [3]). (C) EPZ033294 (green) has a unique binding mode compared to known SMYD2 inhibitors BAY598 (cyan; PDB 5ARG [2]), A893 (magenta; PDB 4YND [9]), LLY507 (yellow; PDB 4WUY [10]), and AZ505 (orange; PDB 3S7B[3]).

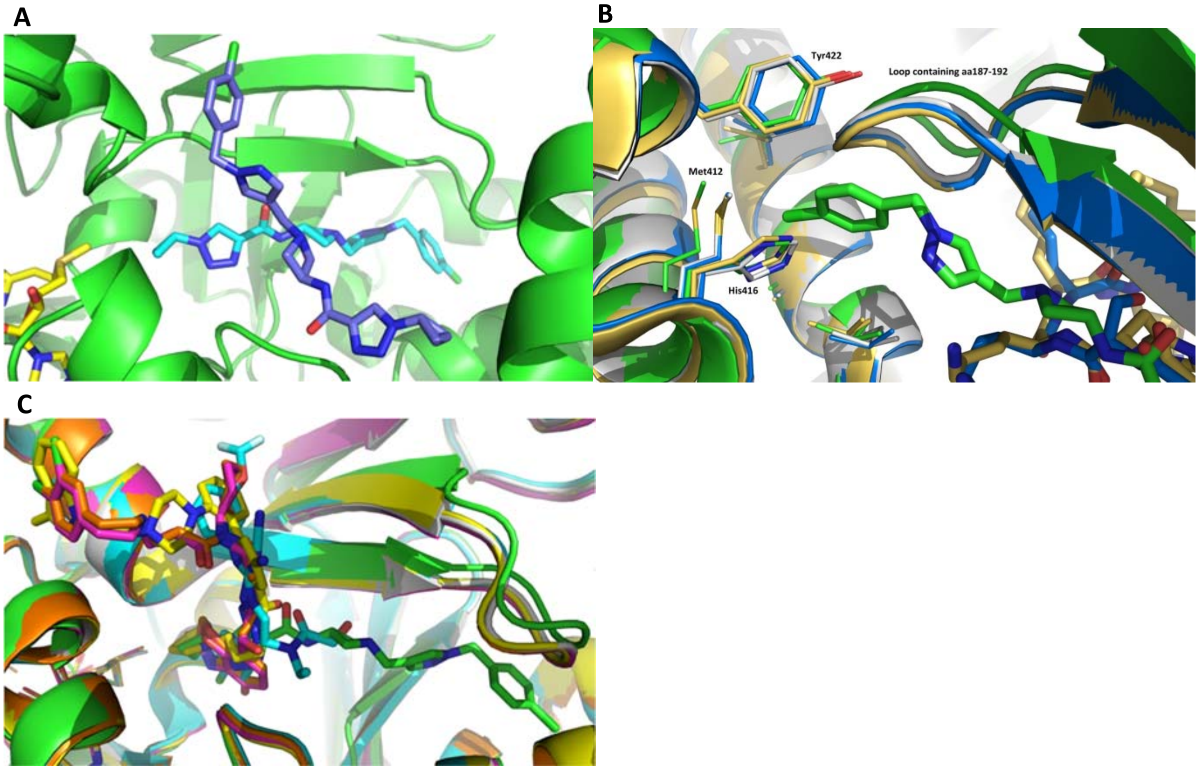

Supplement: S1 Fig — (A) Two molecules of EPZ033294 (molecule 1 = cyan; molecule 2 = purple) were seen in the peptide binding site of SMYD2 (green). SAM (yellow) is show in stick representation. (B) The tail of EPZ033294 molecule 1(green) induces a hydrophobic pocket when compared to structures of SMYD2 (grey; PDB 3TG4 [47]); SMYD2-ERα peptide (yellow; PDB 4O6F [48]) and SMYD2-p53 peptide (blue; PDB 3S7F [13]). (C) EPZ033294 (green) has a unique binding mode compared to known SMYD2 inhibitors BAY598 (cyan; PDB 5ARG [10]), A893 (magenta; PDB 4YND [12]), LLY507 (yellow; PDB 4WUY [11]), and AZ505 (orange; PDB 3S7B[13]). (PDF) [file pone.0197372.s002.pdf]
