# Supplementary material for: Small molecule inhibitors and CRISPR/Cas9 mutagenesis demonstrate that SMYD2 and SMYD3 activity are dispensable for autonomous cancer cell proliferation
Source: PLoS One. 2018 Jun 1;13(6):e0197372. doi: 10.1371/journal.pone.0197372 (PMC5983452; doi:10.1371/journal.pone.0197372)

Figure S2: Scatter plot showing mRNA expression of SMYD2 does not correlate with the IC50 value of LLY507. IC50 values for individual cell lines shown in Fig 3D were plotted against SMYD2 mRNA expression (obtained from the Cell Line Encyclopedia database. (https://portals.broadinstitute.org/ccle)

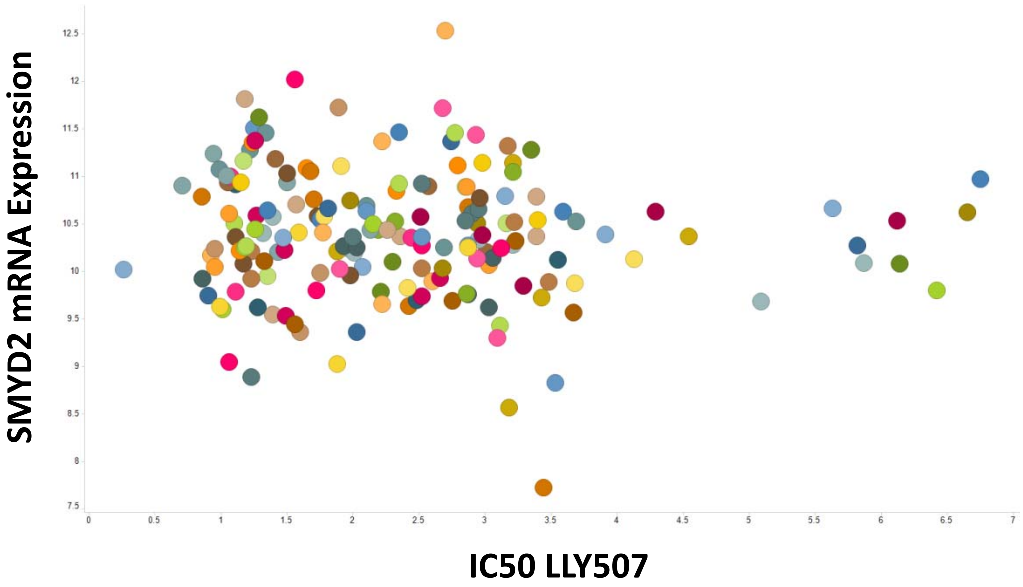

Supplement: S2 Fig — (PDF) [file pone.0197372.s003.pdf]
