# Supplementary material for: Small molecule inhibitors and CRISPR/Cas9 mutagenesis demonstrate that SMYD2 and SMYD3 activity are dispensable for autonomous cancer cell proliferation
Source: PLoS One. 2018 Jun 1;13(6):e0197372. doi: 10.1371/journal.pone.0197372 (PMC5983452; doi:10.1371/journal.pone.0197372)

Figure S3: CETSA with EPZ028862 confirms cellular target engagement. A) Representative western blot showing thermal stability of SMYD3 with and without 100 micromolar EPZ028862. Largest thermal shift with and without EPZ028862 was observed at 47 degrees C. B) Dose-response SMYD3 CETSA for EPZ028862. CETSA EC50 of EPZ028862 at 47 degrees is approximately 1.4 µM. (Representative of 3 western blots).

A

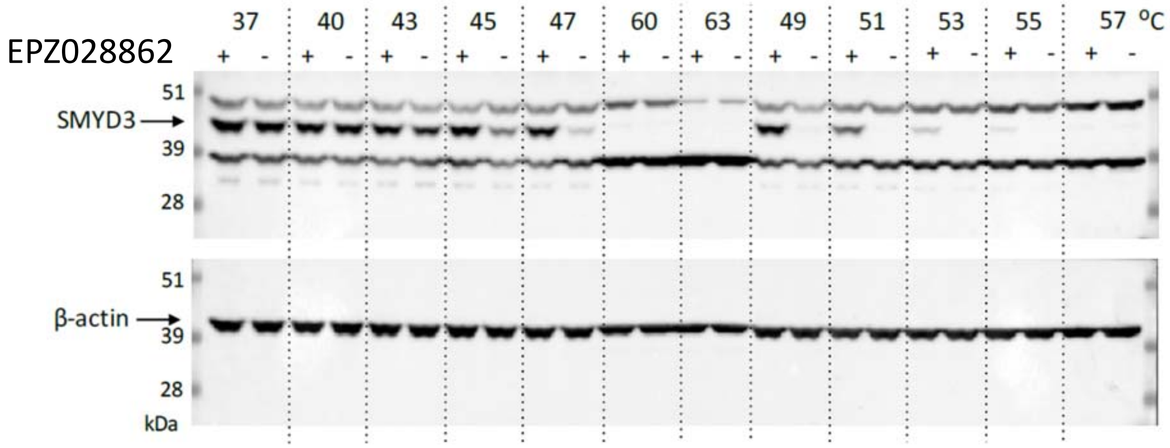

B

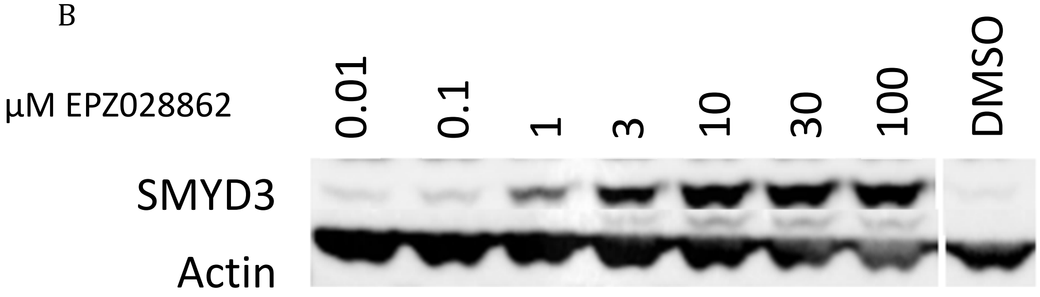

Supplement: S3 Fig — A) Representative western blot showing thermal stability of SMYD3 with and without 100 micromolar EPZ028862. Largest thermal shift with and without EPZ028862 was observed at 47 degrees C. B) Dose-response SMYD3 CETSA for EPZ028862. CETSA EC50 of EPZ028862 at 47 degrees is approximately 1.4 μM. (Representative of 3 western blots). (PDF) [file pone.0197372.s004.pdf]
