# Supplementary material for: Small molecule inhibitors and CRISPR/Cas9 mutagenesis demonstrate that SMYD2 and SMYD3 activity are dispensable for autonomous cancer cell proliferation
Source: PLoS One. 2018 Jun 1;13(6):e0197372. doi: 10.1371/journal.pone.0197372 (PMC5983452; doi:10.1371/journal.pone.0197372)

Figure S4: SMYD3 inhibitor treatment does not affect IC50 of trametinib. A549 cells were treated with varying concentrations of trametinib alone or in combination with 1 μM EPZ028862 for 2, 5 and 7 days. Addition of EPZ028862 has no effect on growth inhibition by trametinib in A549 cells. Plotted data is the average of three biological replicates. Error bars represent standard deviation.

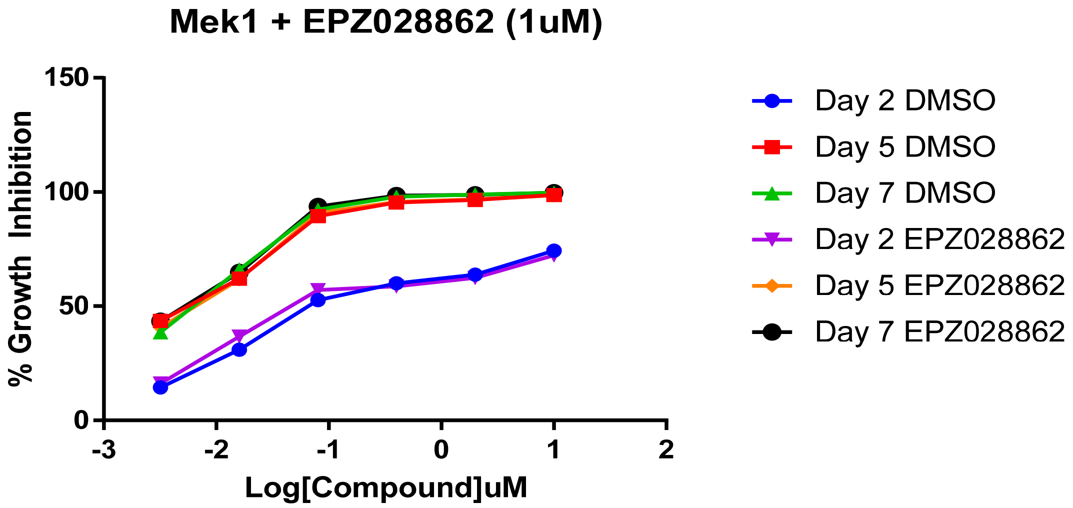

Supplement: S4 Fig — A549 cells were treated with varying concentrations of trametinib alone (left) or in combination with 1 µM EPZ028862(right) for 2, 5 and 7 days. Addition of EPZ028862 has no effect on growth inhibition by trametinib in A549 cells. Plotted data is the average of three biological replicates. Error bars represent standard deviation. (PDF) [file pone.0197372.s005.pdf]
