# Supplementary material for: Small molecule inhibitors and CRISPR/Cas9 mutagenesis demonstrate that SMYD2 and SMYD3 activity are dispensable for autonomous cancer cell proliferation
Source: PLoS One. 2018 Jun 1;13(6):e0197372. doi: 10.1371/journal.pone.0197372 (PMC5983452; doi:10.1371/journal.pone.0197372)

Figure S5.CRISPR pooled screen data for 313 cell lines for two pan-essential controls,PLK1 (A) and EIF4A3 (B). On the y-axis is the sensitivity p-value (LogP RSA) for the indicated target gene. Each bar represents one of 313 cell lines.

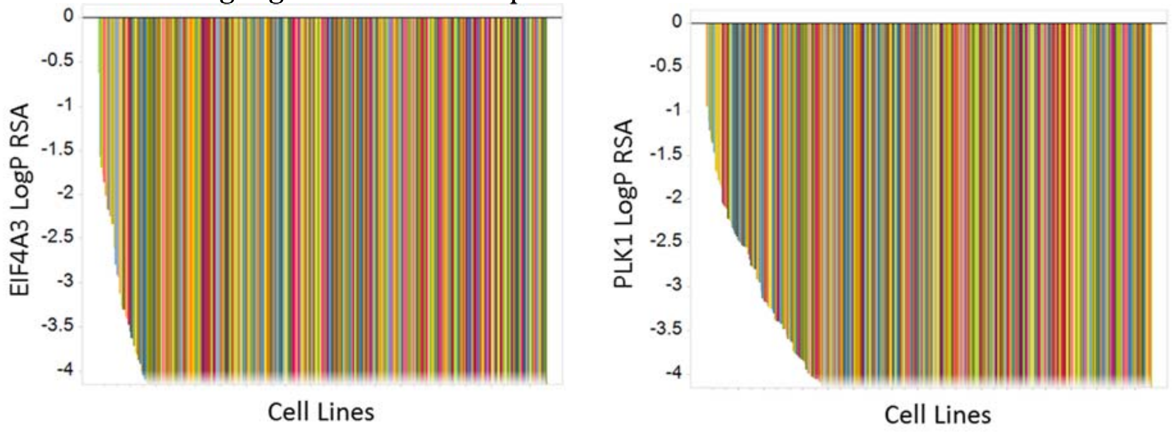

Supplement: S5 Fig — CRISPR pooled screen data for 313 cell lines for two pan-essential controls, PLK1 (A) and EIF4A3 (B). On the y-axis is the sensitivity p-value. (PDF) [file pone.0197372.s006.pdf]
