# Supplementary material for: Small molecule inhibitors and CRISPR/Cas9 mutagenesis demonstrate that SMYD2 and SMYD3 activity are dispensable for autonomous cancer cell proliferation
Source: PLoS One. 2018 Jun 1;13(6):e0197372. doi: 10.1371/journal.pone.0197372 (PMC5983452; doi:10.1371/journal.pone.0197372)

Figure S6: Growth of SNU-475 and SNU-423 cell lines were evaluated following SMYD3 knockout. A and C show Incucyte growth curves of both cell lines with virus containing a sgRNA targeting the fetal hemoglobin gene (HBE1) or exon 2 of SMYD3. Plotted data is the average of three biological replicates. Error bars represent standard deviation. B and D confirm persistent knockout of SMYD3 in SMYD3 sgRNA infected cells out to 19 days.

A

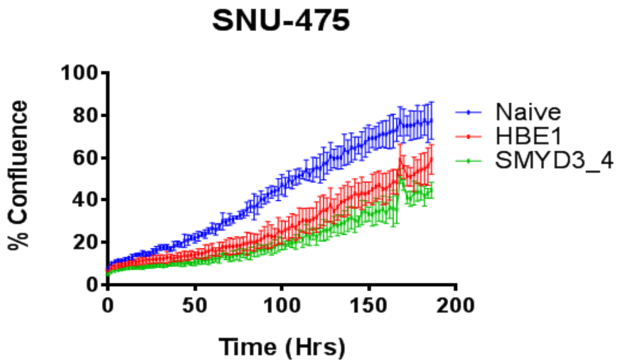

B

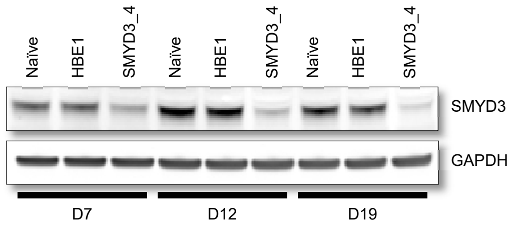

C

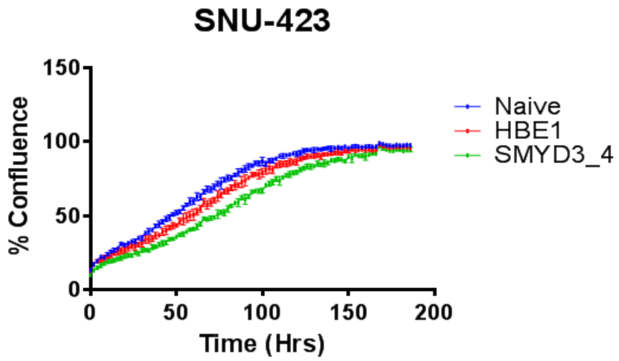

D

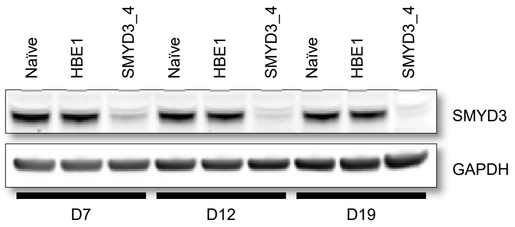

Supplement: S6 Fig — A and C show Incucyte growth curves of both cell lines with virus containing a sgRNA targeting the fetal hemoglobin gene (HBE1) or exon 2 of SMYD3. Plotted data is the average of three biological replicates. Error bars represent standard deviation. B and D confirm persistent knockout of SMYD3 in SMYD3 sgRNA infected cells out to 19 days. (PDF) [file pone.0197372.s007.pdf]
