# Supplementary material for: Small molecule inhibitors and CRISPR/Cas9 mutagenesis demonstrate that SMYD2 and SMYD3 activity are dispensable for autonomous cancer cell proliferation
Source: PLoS One. 2018 Jun 1;13(6):e0197372. doi: 10.1371/journal.pone.0197372 (PMC5983452; doi:10.1371/journal.pone.0197372)

Figure S7: Development of BTF3K1me1 antibody. Serum from rabbits injected with adjuvant conjugated peptide corresponding to methylated K1 of BTF3 (K(Me)-ETIMNQEKLAKC) was tested for activity by western blot. Lysates from 293T cells overexpressing SMYD2 or KYSE-150 cells treated with increasing concentrations of LLY-507 were collected. Western blot analysis was performed using affinity purified anti-BTF3me1 antibody. Cells over-expressing SMYD2 show an increase in anti-BTF3me1 signal. Cells treated with LLY-507 show a decrease in anti-BTF3me1 signal.

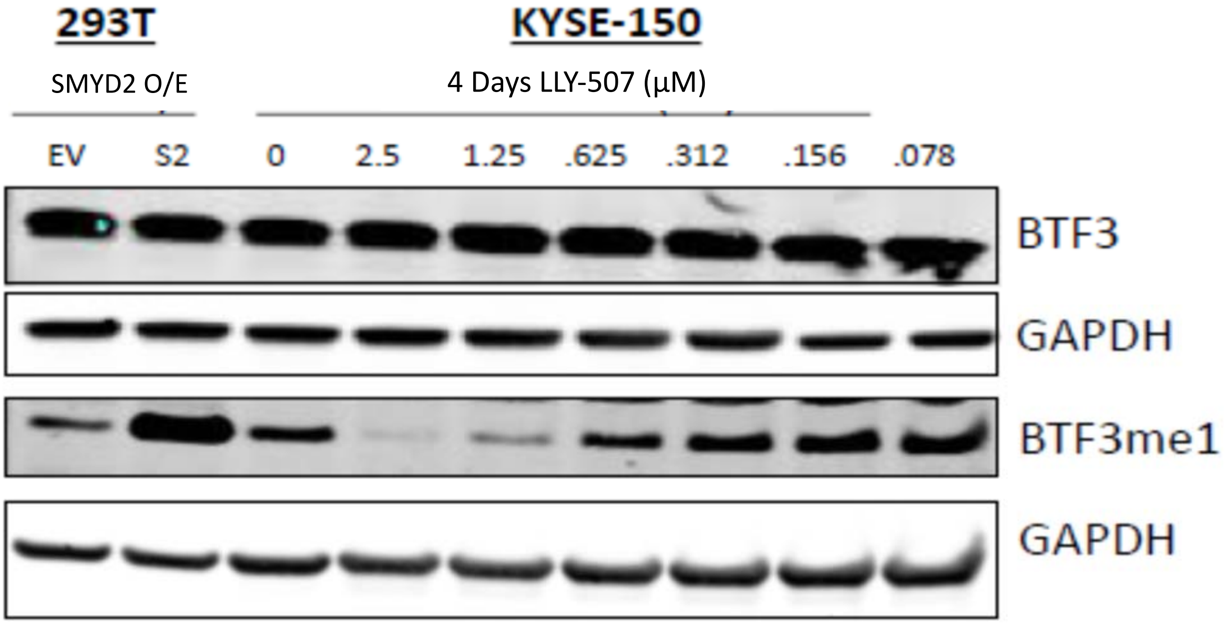

Supplement: S7 Fig — Serum from rabbits injected with adjuvant conjugated peptide corresponding to methylated K1 of BTF3 (K(Me)-ETIMNQEKLAKC) was tested for activity by western blot. Lysates from 293T cells overexpressing SMYD2 or KYSE-150 cells treated with increasing concentrations of LLY-507 were collected. Western blot analysis was performed using affinity purified anti-BTF3me1 antibody. Cells over-expressing SMYD2 show an increase in anti-BTF3me1 signal. Cells treated with LLY-507 show a decrease in anti-BTF3me1 signal. (PDF) [file pone.0197372.s008.pdf]
