# Supplementary material for: Small molecule inhibitors and CRISPR/Cas9 mutagenesis demonstrate that SMYD2 and SMYD3 activity are dispensable for autonomous cancer cell proliferation
Source: PLoS One. 2018 Jun 1;13(6):e0197372. doi: 10.1371/journal.pone.0197372 (PMC5983452; doi:10.1371/journal.pone.0197372)

Figure S8. SMYD2 substrate steady-state kinetics. Initial velocities with their standard error from timecourse data in duplicate are shown as function of substrate concentration. Rates for varied peptide at 2 nM SMYD2 and 50 nM SAM were fit using eq 1 which gives a K_M value for H3,1-29 of 66 ± 11 nM from 1 experiment (A). Rates for varied SAM at 1 nM SMYD2 and 60 nM H3,1-29 were fit using eq 2 which gives a K_M value for SAM of 0.34 ± 0.07 nM from 1 experiment (B).

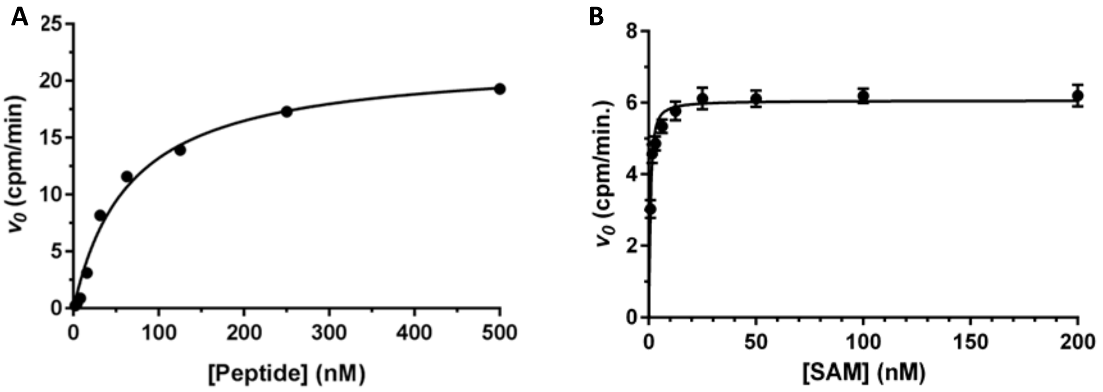

Supplement: S8 Fig — Initial velocities with their standard error from timecourse data in duplicate are shown as function of substrate concentration. Rates for varied peptide at 2 nM SMYD2 and 50 nM SAM were fit using eq 1 which gives a KM value for H3,1–29 of 66 ± 11 nM from 1 experiment (A). Rates for varied SAM at 1 nM SMYD2 and 60 nM H3,1–29 were fit using Eq 2 which gives a KM value for SAM of 0.34 ± 0.07 nM from 1 experiment (B). (PDF) [file pone.0197372.s009.pdf]
