# Supplementary material for: Small molecule inhibitors and CRISPR/Cas9 mutagenesis demonstrate that SMYD2 and SMYD3 activity are dispensable for autonomous cancer cell proliferation
Source: PLoS One. 2018 Jun 1;13(6):e0197372. doi: 10.1371/journal.pone.0197372 (PMC5983452; doi:10.1371/journal.pone.0197372)

Figure S9. Mechanism of inhibition of SMYD2 by EPZ032597. IC_{50} values with their standard error from eq 4 are plotted as a function of peptide concentration. EPZ032597 inhibition is best described as noncompetitive versus peptide using eq 6 with a K_i value of 21.5 ± 1.5 nM from one experiment.

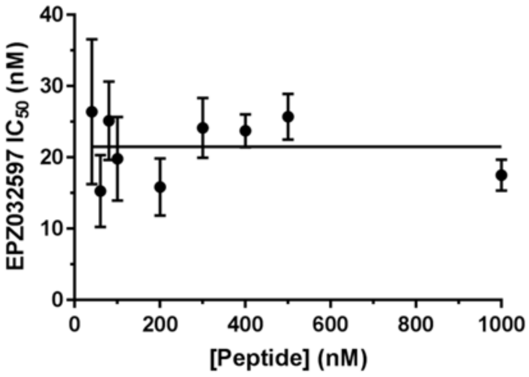

Supplement: S9 Fig — IC50 values with their standard error from Eq 4 are plotted as a function of peptide concentration. EPZ032597 inhibition is best described as noncompetitive versus peptide using Eq 6 with a Ki value of 21.5 ± 1.5 nM from one experiment. (PDF) [file pone.0197372.s010.pdf]
