# Supplementary material for: Small molecule inhibitors and CRISPR/Cas9 mutagenesis demonstrate that SMYD2 and SMYD3 activity are dispensable for autonomous cancer cell proliferation
Source: PLoS One. 2018 Jun 1;13(6):e0197372. doi: 10.1371/journal.pone.0197372 (PMC5983452; doi:10.1371/journal.pone.0197372)

Figure S10: Biophysical characterization of EPZ033294 to SMYD2. (A) One representative thermogram for ITC binding of EPZ033294 to SMYD2 is shown. Stoichiometry of binding in this experiment was found to be 0.7. (B) Measurement of binding of EPZ033294 to SMYD2 by SPR assay. The dissociation constant (K_D) was determined to be 5 nM, with a k_{on} = 5 x 10^5 $M^{-1}s^{-1}$ and k_{off} = 0.003 s^{-1}.

A

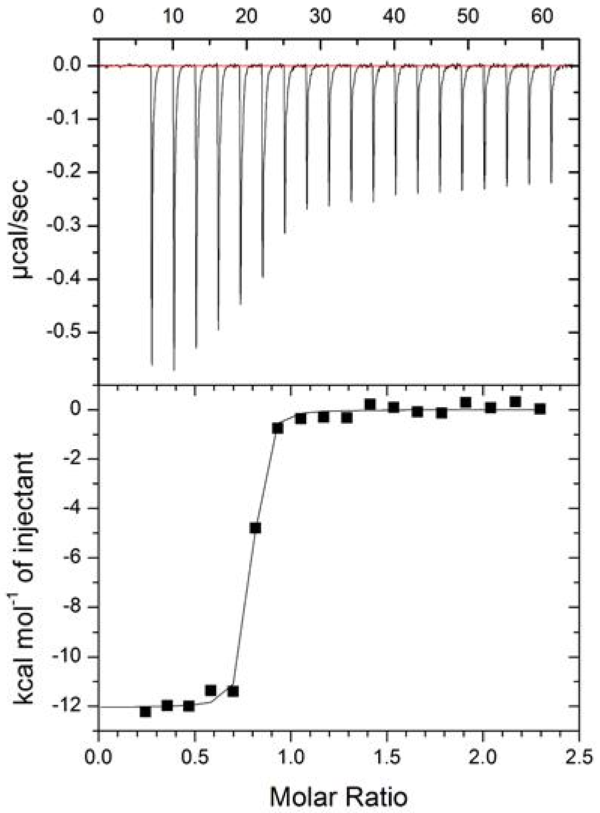

B

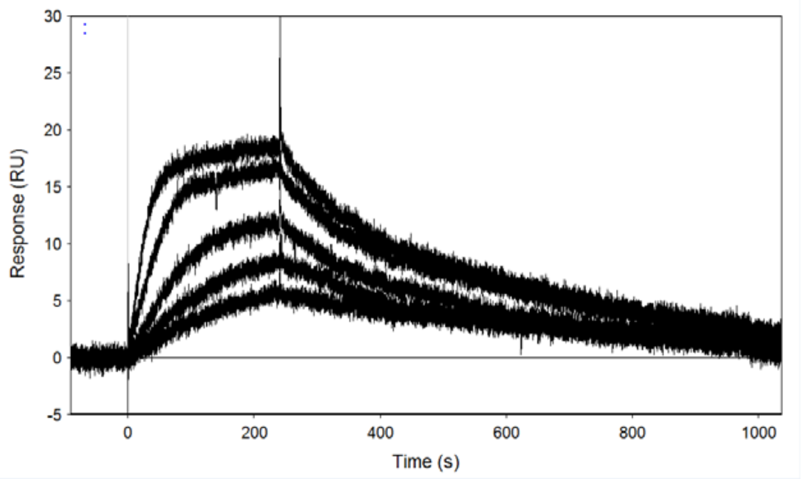

Supplement: S10 Fig — (A) One representative thermogram for ITC binding of EPZ033294 to SMYD2 is shown. Stoichiometry of binding in this experiment was found to be 0.7. (B) Measurement of binding of EPZ033294 to SMYD2 by SPR assay. The dissociation constant (KD) was determined to be 5 nM, with a kon = 5 x 10^5^ M-1s-1 and koff = 0.003 s-1. (PDF) [file pone.0197372.s011.pdf]
