# Supplementary material for: Small molecule inhibitors and CRISPR/Cas9 mutagenesis demonstrate that SMYD2 and SMYD3 activity are dispensable for autonomous cancer cell proliferation
Source: PLoS One. 2018 Jun 1;13(6):e0197372. doi: 10.1371/journal.pone.0197372 (PMC5983452; doi:10.1371/journal.pone.0197372)

Figure S11. Mechanism of inhibition of SMYD3 by EPZ028862. EPZ028862 IC_{50} values with their standard error from eq 4 are plotted as a function of MEKK2 (A) and SAM (B) concentration using the filterplate assay. EPZ028862 inhibition is best described as noncompetitive versus MEKK2 (eq 6) and mixed-type inhibition versus SAM (eq 5). Values for the inhibition constants are shown in Table S3.

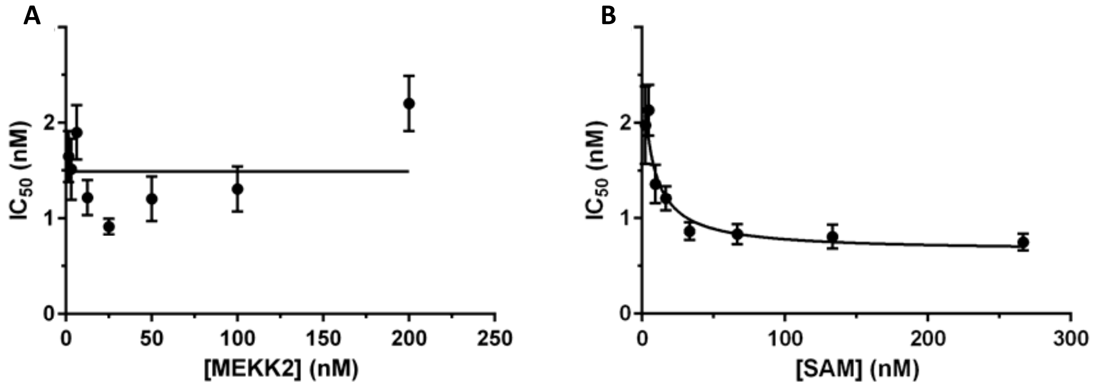

Supplement: S11 Fig — EPZ028862 IC50 values with their standard error from Eq 4 are plotted as a function of MEKK2 (A) and SAM (B) concentration using the filterplate assay. EPZ028862 inhibition is best described as noncompetitive versus MEKK2 (Eq 6) and mixed-type inhibition versus SAM (Eq 5). Values for the inhibition constants are shown in S3 Table. (PDF) [file pone.0197372.s012.pdf]
