# Supplementary material for: Small molecule inhibitors and CRISPR/Cas9 mutagenesis demonstrate that SMYD2 and SMYD3 activity are dispensable for autonomous cancer cell proliferation
Source: PLoS One. 2018 Jun 1;13(6):e0197372. doi: 10.1371/journal.pone.0197372 (PMC5983452; doi:10.1371/journal.pone.0197372)

Figure S12: Binding of EPZ028862 to SMYD3 by SPR. A single representative sensogram (red) is shown with the calculated fit (black). Kinetic constants k_{on} ($6.1 \pm 1.1 \times 10^5\ M^{-1}s^{-1}$) and k_{off}, ($2.1 \pm 0.57 \times 10^{-4}\ s^{-1}$) were based on fitted values from seven replicate measurements (mean ± standard deviation).

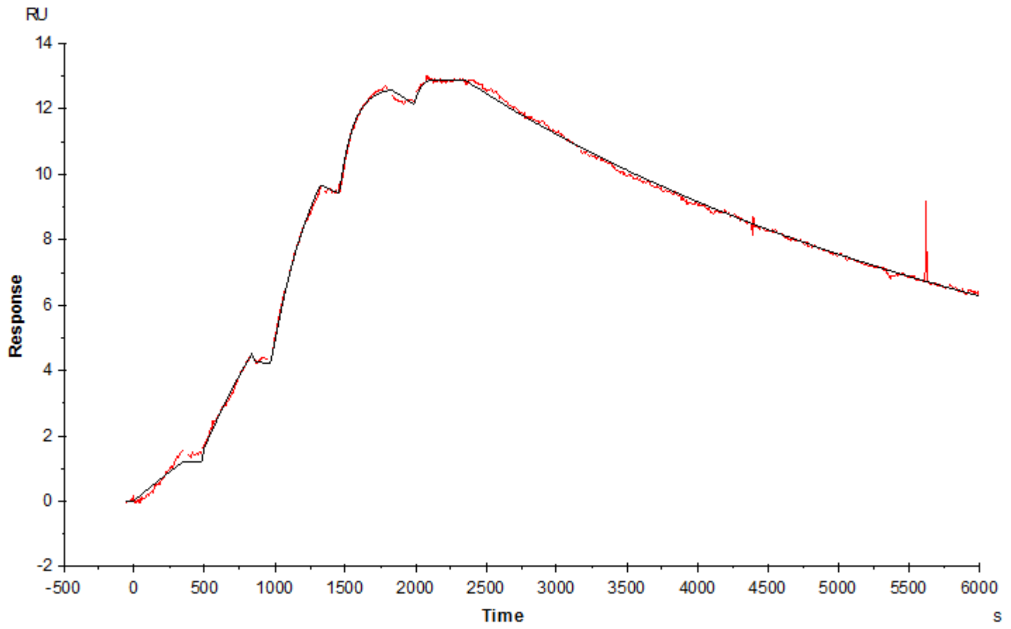

Supplement: S12 Fig — A single representative sensogram (red) is shown with the calculated fit (black). Kinetic constants kon (6.1 ± 1.1 x 10^5^ M-1s-1) and koff, (2.1 ± 0.57 x 10−4 s-1) were based on fitted values from seven replicate measurements (mean ± standard deviation). (PDF) [file pone.0197372.s013.pdf]
