# Supplementary material for: Small molecule inhibitors and CRISPR/Cas9 mutagenesis demonstrate that SMYD2 and SMYD3 activity are dispensable for autonomous cancer cell proliferation
Source: PLoS One. 2018 Jun 1;13(6):e0197372. doi: 10.1371/journal.pone.0197372 (PMC5983452; doi:10.1371/journal.pone.0197372)

Figure S13: Superposition of EPZ028862 (cyan) and EPZ030456 (grey; PDB 5CCM [4]) shows the overall binding mode of the compounds in the SMYD3 (green) binding site is similar despite the different groups bound in the lysine channel and differing tail moieties. SAM (yellow) is shown in stick representation.

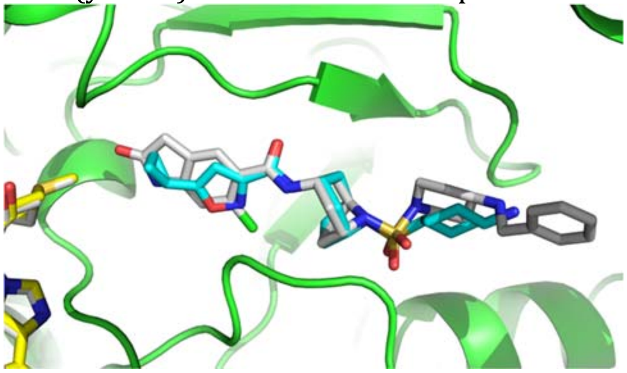

Supplement: S13 Fig — SAM (yellow) is shown in stick representation. (PDF) [file pone.0197372.s014.pdf]
