# Supplementary material for: Small molecule inhibitors and CRISPR/Cas9 mutagenesis demonstrate that SMYD2 and SMYD3 activity are dispensable for autonomous cancer cell proliferation
Source: PLoS One. 2018 Jun 1;13(6):e0197372. doi: 10.1371/journal.pone.0197372 (PMC5983452; doi:10.1371/journal.pone.0197372)
Supplement: S1 Table — All reported results for off-target enzymes were tested in duplicate. (PDF) [file pone.0197372.s015.pdf]

Table S1: Protein methylatransferase selectivity panel. All reported results for off-target enzymes were tested in duplicate. On-target values are as reported in Table 1.

Target	EPZ-028862-7	EPZ-032597-2	EPZ-033294-1
EHMT1	>10uM	>10uM	>10uM
EHMT2	>10uM	>10uM	>10uM
EZH1	>10uM	>10uM	>10uM
EZH2	>10uM	>10uM	>10uM
NSD1	>10uM	>10uM	>10uM
PRDM9	>10uM	>10uM	>10uM
PRMT3	>10uM	>10uM	>10uM
PRMT6	>10uM	>10uM	>10uM
PRMT7	>10uM	>10uM	>10uM
PRMT8	>10uM	>10uM	>10uM
SETD2	>10uM	>10uM	>10uM
SETD7	>10uM	>10uM	>10uM
SMYD2	>10uM	16 nM	3.9 nM
SMYD3	1.8 nM	>10uM	>10uM
SUV39H1	>10uM	>10uM	>10uM
WHSC1	>10uM	>10uM	>10uM
