# Supplementary material for: Small molecule inhibitors and CRISPR/Cas9 mutagenesis demonstrate that SMYD2 and SMYD3 activity are dispensable for autonomous cancer cell proliferation
Source: PLoS One. 2018 Jun 1;13(6):e0197372. doi: 10.1371/journal.pone.0197372 (PMC5983452; doi:10.1371/journal.pone.0197372)
Supplement: S2 Table — (PDF) [file pone.0197372.s016.pdf]

Table S2. Crystallographic data collection and refinement statistics for SMYD2 and SMYD3 crystal structures

	SMYD2-EPZ033294	SMYD3-EPZ028862
**Data collection**		
Space group	C2	$P2_12_12_1$
Cell dimensions		
a, *b*, *c* (Å)	157.6, 54.8, 79.7	60.8, 65.9, 107.0
α, β, γ (°)	90.0, 114.2, 90.0	90.0, 90.0, 90.0
Resolution (Å)	50.00-2.69(2.74-2.69)	17.26-1.42 (1.45-1.42)
R_{sym} or R_{merge}	0.089 (0.588)	0.074 (0.346)
$I / \sigma I$	14.6 (2.4)	8.8 (2.6)
Completeness (%)	98.8 (100)	97.7 (85.9)
Redundancy	3.7 (3.8)	5.8 (4.1)
**Refinement**		
Resolution (Å)	50-2.69	56.09-1.42
No. reflections	16191	75322
R_{work} / R_{free}	0.218/0.270	0.228/0.279
No. atoms		
Protein	3535	3689
EPZ/SAM/Non-water solvent/Zn	58/27/30/3	29/27/27/3
Water	129	499
B-factors		
Protein	55.3	17.2
EPZ/SAM/Non-water solvent/Zn	37.2/46.4/47.3/29.3	11.1/9.5/34.5/13.0
Water	36.9	27.0
R.m.s. deviations		
Bond lengths (Å)	0.010	0.008
Bond angles (°)	1.345	1.351

1 crystal was used for each structure. *Values in parentheses are for highest-resolution shell.
