# Supplementary material for: Small molecule inhibitors and CRISPR/Cas9 mutagenesis demonstrate that SMYD2 and SMYD3 activity are dispensable for autonomous cancer cell proliferation
Source: PLoS One. 2018 Jun 1;13(6):e0197372. doi: 10.1371/journal.pone.0197372 (PMC5983452; doi:10.1371/journal.pone.0197372)
Supplement: S3 Table — (PDF) [file pone.0197372.s017.pdf]

Table S3: *In Vitro* and *In Vivo* DMPK Results

Study	Parameters/Species	EPZ028862	EPZ033294
Caco-2 Permeability *	$P_{app,AB}$ (10^{-6} cm/s)	0.46, 0.64	2.18
	$P_{app,BA}$ (10^{-6} cm/s)	16.4, 13.7	64.3
	Efflux Ratio	36, 21	29
Plasma protein binding * (expressed as unbound fraction f_u)	Mouse f_u	0.51	0.26
	Rat f_u	0.68, 0.6	
	Dog f_u	0.82	
	Monkey f_u	Highly bound	
	Human f_u	0.68, 0.73	0.13
CL_{int} measured with liver microsomes * (µL/min/mg microsomal protein)	Mouse	3.3	
	Rat	<3, 3.4	
	Dog	<3	
	Monkey	3.2	
	Human	<3, 3.3	
CL_{int} measured with hepatocytes * (µL/min/10^6 cells)	Mouse	6.6	10.9
	Rat	5.2, 3.5	
	Dog	<3	
	Monkey	3.2	
	Human	4.8, 3.7, <3	6.0
Mouse PK ^ Male CD-1*For EPZ028862*IV: 1 mg/kg (saline, pH 4)PO: 5 mg/kg (saline, pH 4)Terminal bleeding*For EPZ033294*IV: 2 mg/kg (10:90 ethanol:saline)PO: 10 mg/kg (10:90 ethanol:saline)Serial bleeding	CLp (mL/min/kg)	79.8	37.4 ± 6.7
	CLr (mL/min/kg)	23.6	ND
	Vss (L/kg)	9.5	4.5 ± 1.0
	$t_{1/2}$ (hr)	2.0	3.2 ± 1.0
	MRT (hr)	2.0	1.8 ± 0.3
	t_{max} (hr)	0.33	1.7 ± 0.6
	F (%)	~100	241 ± 20
Rat PK ^Male, Sprague DawleyIV: 1 mg/kg (saline, pH 4)PO: 5 mg/kg (saline, pH 4)Serial bleeding	CLp (mL/min/kg)	32.7 ± 4.4	
	CLr (mL/min/kg)	21 ± 6	
	Vss (L/kg)	4.4 ± 0.1	
	$t_{1/2}$ (hr)	2.1 ± 0.2	
	MRT (hr)	59 ± 10	
	t_{max} (hr)	1.0^+	
	F (%)	59 ± 10	
Dog PK ^Male, Beagle IV: 1 mg/kg (5:95 ethanol:saline)PO: 5 mg/kg (0.1% Tween in 0.5% methylcellulose water solution)Serial bleeding	CLp (mL/min/kg)	13.9 ± 1.2	
	CLr (mL/min/kg)	1.97 ± 0.38	
	Vss (L/kg)	5.8 ± 0.6	
	$t_{1/2}$ (hr)	5.4 ± 0.1	
	MRT (hr)	7.0 ± 0.2	
	t_{max} (hr)	1.5 ± 0.9	
	F (%)	70 ± 6	
NHP PK ^Male, Cynomolgus IV: 1 mg/kg (5% ethanol aqueous solution)PO: 5 mg/kg (0.1% Tween in 0.5% methylcellulose water solution)Serial bleeding	CLp (mL/min/kg)	21.4 ± 3.4	
	CLr (mL/min/kg)	1.06 ± 0.84	
	Vss (L/kg)	9.5 ± 2.5	
	$t_{1/2}$ (hr)	5.6 ± 1.3	
	MRT (hr)	7.4 ± 1.5	
	t_{max} (hr)	2.0^+	
	F (%)	62 ± 13	

*Values are the mean of duplicates within each experiment; values for individual experiments reported separately^ Results are expressed as Mean +/- standard deviation (n=3) for studies performed with serial bleeding. For results from non-serial bleeding methods, the mean plasma concentration of each timepoint (n=3) was used for PK parameter calculation and a single value is reported.$^+$All three animals showed the same t_{max} value.
