# Supplementary material for: Small molecule inhibitors and CRISPR/Cas9 mutagenesis demonstrate that SMYD2 and SMYD3 activity are dispensable for autonomous cancer cell proliferation
Source: PLoS One. 2018 Jun 1;13(6):e0197372. doi: 10.1371/journal.pone.0197372 (PMC5983452; doi:10.1371/journal.pone.0197372)
Supplement: S4 Table — (PDF) [file pone.0197372.s018.pdf]

Table S4. SMYD3 Inhibition constants for EPZ028862.

Parameter	varied substrate MEKK2[1]	SAM[2]
Mechanism	noncompetitive	mixed
K_i (nM)	1.49 ± 0.15	2.64 ± 0.48
αK_i (nM)	NA[3]	0.65 ± 0.13
α	NA[3]	0.25 ± 0.05

[1]Fit value and standard error from fit to eq 6 above from one experiment. SMYD3 and SAM concentrations of 0.08 and 8 nM respectively were used.[2]Fit value and standard error from fit to eq 5 above from one experiment. SMYD3 and MEKK2 concentrations of 0.39 and 12 nM respectively were used.[3]NA = not applicable
