# Supplementary material for: Small molecule inhibitors and CRISPR/Cas9 mutagenesis demonstrate that SMYD2 and SMYD3 activity are dispensable for autonomous cancer cell proliferation
Source: PLoS One. 2018 Jun 1;13(6):e0197372. doi: 10.1371/journal.pone.0197372 (PMC5983452; doi:10.1371/journal.pone.0197372)
Supplement: S5 Table — (PDF) [file pone.0197372.s019.pdf]

Table S5. Proliferation IC50s and KRAS mutant status of assorted lung cancer cell lines.

Cell Line	Histology	KRAS Mutation (CCLE)	IC_{50}
A549	lung, carcinoma	G12S	>20
NCI-H23	lung, non-small cell lung cancer	G12C	>20
NCI-H441	lung, papillary adenocarcinoma	G12V	>20
NCI-H2122	lung, non-small cell lung cancer	G12C	>20
SK-LU-1	lung, adenocarcinoma	G12D	>20
COR-L105	lung, adenocarcinoma	WT	>20
SW1573	lung, alveolar cell carcinoma	G12C	>20
SHP77	lung, small cell lung cancer	G12V	>20
DMS53	lung, small cell lung cancer	WT	>20
DMS153	lung, small cell lung cancer	Unk.	>20
COR-L88	lung, small cell carcinoma	WT	>20
NCI-H2029	lung, small cell lung cancer	WT	>20
NCI-H2081	lung, small cell lung carcinoma	WT	>20
